# Association of organ dysfunction trajectories and major adverse cardiovascular events using clinical obesity in UK Biobank

**DOI:** 10.3389/fendo.2026.1844870

**Published:** 2026-05-14

**Authors:** Yohwan Lim, Su Kyoung Lee

**Affiliations:** 1 Gochang-gun Public Health Center, Ministry of Health and Welfare, Go-Chang, Republic of Korea; 2 Department of Healthcare Management, College of Health and Medical Science, Daejeon University, Daejeon, Republic of Korea

**Keywords:** body mass index, clinical obesity, major adverse cardiovascular events, obesity, trajectory

## Abstract

**Objective:**

Obesity is a major modifiable risk factor for cardiovascular disease. We evaluated whether multi-organ dysfunction trajectories for major adverse cardiovascular events (MACE) using clinically defined obesity classification improves risk discrimination compared with BMI-based categories.

**Methods:**

We analyzed 457,675 UK Biobank participants. BMI-based categories were defined using ethnicity-specific thresholds. Clinically defined obesity was classified as no, preclinical (excess adiposity without organ dysfunction), or clinical obesity (excess adiposity with ≥1 obesity-related organ dysfunction). Organ dysfunction trajectories were constructed over consecutive 2-year periods beginning 2 years after baseline: (1) status (no, period 1 only, period 2 only, persistent organ dysfunctions) and (2) change (no, increase, decrease, persistent organ dysfunctions). Incident MACE was defined as a composite of ischemic heart disease, stroke, and fatal cardiovascular disease. Participants were followed until the first occurrence of incident MACE, death, 10 years after the index date, or the end of follow up, whichever came first. Cox models estimated adjusted hazard ratio (aHR).

**Results:**

Across status trajectories, MACE risk increased in a graded response from no dysfunction to persistent dysfunction under both obesity definitions. Within trajectories, clinical obesity showed the highest risk under the clinically defined definition (clinical vs no obesity: no dysfunction aHR 2.03, 95% CI 1.96–2.11; persistent dysfunction aHR 1.61, 95% CI 1.30–2.01), whereas underweight showed the highest risk under BMI categories (underweight vs normal: no dysfunction aHR 1.83, 95% CI 1.62–2.07; persistent dysfunction aHR 2.19, 95% CI 1.32–3.63). The 10-year cumulative incidence of MACE within persistent dysfunction increased monotonically across clinically defined categories (~24% in clinical obesity), while BMI-based categories peaked in the overweight (~20%).

**Conclusions:**

Multi-organ dysfunction trajectories were strongly associated with incident MACE, having persistent dysfunction as the highest risk. Clinically defined obesity provided more monotonic, graded risk across trajectories than BMI, which were susceptible to reverse causation. Incorporating clinical obesity definitions with longitudinal dysfunction assessment may improve cardiovascular risk stratification.

## Introduction

Obesity is a major modifiable risk factor for cardiovascular morbidity and mortality, yet risk stratification based on body mass index (BMI) remains imperfect (1). BMI does not distinguish fat from lean mass, cannot capture fat distribution, and may misclassify individuals with preserved metabolic and organ function as high risk while overlooking those with substantial obesity-related organ impairment despite lower BMI (2–4). These limitations have motivated new approaches that conceptualize obesity as a systemic chronic disease characterized not only by excess adiposity but also by organ dysfunction.

The 2025 Lancet Diabetes & Endocrinology Commission proposed a clinically oriented definition that distinguishes preclinical obesity (excess adiposity without any organ dysfunction) from clinical obesity (excess adiposity with objective organ dysfunction or substantial functional limitation), redefining obesity as a chronic, systemic disease rather than body size alone (5). Early studies of this definition have shown that clinical obesity identifies individuals at substantially higher longitudinal risk of diabetes, cardiovascular events, and mortality compared with those without obesity or without dysfunction, and that organ dysfunction itself may carry meaningful prognostic information, independent of obesity (6, 7). In addition, Xu et al. modeled transitions from preclinical obesity to clinical obesity and adverse outcomes, supporting the clinical relevance of progression in dysfunction states rather than a single cross-sectional assessment (8).

However, most prior studies applying the clinical obesity definition have evaluated dysfunction cross-sectionally and few have assessed whether repeated assessment of dysfunction improves cardiovascular risk stratification. Therefore, using the UK Biobank, we examined whether organ dysfunction trajectories are associated with subsequent major adverse cardiovascular events (MACE) and whether clinically defined obesity definition better captures the risk across dysfunction trajectories compared with BMI-based categories.

## Methods

### Study population

#### UK Biobank cohort

**Figure 1:**
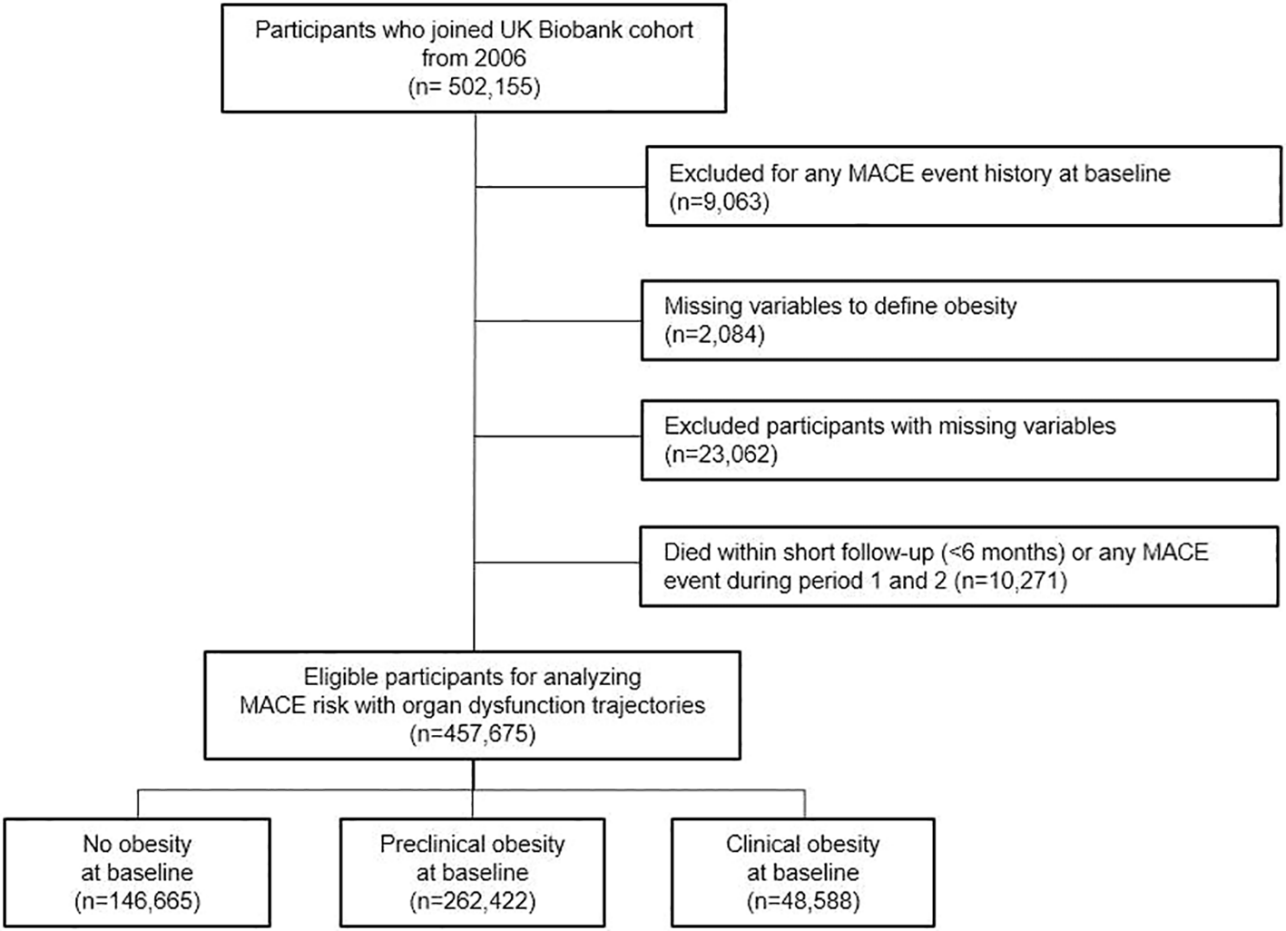
Flow diagram for the inclusion of the study population.

UK Biobank is a large, prospective cohort study of around 500,000 participants recruited from United Kingdom (UK). It comprises extensive data on sociodemographic characteristics, anthropometric measurements, questionnaire data, demographic and lifestyle data, and healthcare records. Participants were enrolled between 2006 and 2010, when baseline characteristics were collected. Within healthcare records, any hospital inpatient data, cancer data, and procedure data are available for every participant. However, the availability of healthcare records varies by its region and data source. Therefore, region-specific administrative censoring dates were applied using Hospital Episode Statistics for England (March 31, 2023), Scottish Morbidity Record (August 31, 2022), and the Patient Episode Database for Wales (May 31, 2022). Among 502,155 participants from UK Biobank dataset, we excluded those with any history of major adverse cardiovascular event (MACE) before follow-up (n=9,063), missing variables to define obesity (n=2,084), missing information for other covariates (n = 23,062), and died within short follow-up (<6 months) or any MACE event during period 1 and 2 (n=10,271) were excluded. Therefore, 457,675 UK Biobank cohort participants were finally enrolled for our analysis (**Figure 1**). Further details of the UK Biobank cohort and data resources are available elsewhere (9). Our study using UK Biobank dataset obtained Research Tissue Bank approval from the North West Multi-centre Research Ethics Committee (ID: 16/NW/0274).

### BMI-based obesity

BMI-based obesity categories were assigned using ethnicity-specific BMI thresholds. Among non-Asian participants, BMI was classified as underweight (<18.5 kg/m²), normal weight (18.5–24.9 kg/m²), overweight (25.0–29.9 kg/m²), and obese (≥30.0 kg/m²). Among Asian participants, BMI was classified as underweight (<18.5 kg/m²), normal weight (18.5–22.9 kg/m²), overweight (23.0–24.9 kg/m²), and obese (≥25.0 kg/m²) (10).

### Clinically defined obesity

Clinically defined obesity was defined as no, preclinical, or clinical obesity, based on excess adiposity with any additional diagnosis of obesity-related organ dysfunction following the new obesity definition by 2025 Lancet Diabetes & Endocrinology Commission (5). Excess adiposity was confirmed using a multi-measure anthropometric definition based on BMI, waist circumference (WC), waist-to-hip ratio (WHR), waist-to-height ratio (WHtR), and body fat percentage (BF). Central adiposity was defined as WC ≥102 cm (men) or ≥88 cm (women) for non-Asian participants and ≥90 cm (men) or ≥80 cm (women) for Asian participants; WHR ≥0.90 (men) or ≥0.85 (women); and WHtR ≥0.50. Elevated BMI was defined as ≥30 kg/m² for non-Asian participants and ≥25 kg/m² for Asian participants, and elevated BF as ≥25% for men and ≥35% for women. Excess adiposity was confirmed if any of the following were met: (1) elevated BF; (2) elevated BMI plus ≥1 elevated central adiposity measure (WC, WHR, or WHtR); or (3) ≥2 elevated central adiposity measures (10). Obesity-related organ dysfunction was defined using hospital inpatient diagnosis by ICD-10 codes, which included organ dysfunctions from central nervous system, upper airway, respiratory, cardiovascular, metabolic, liver, renal, urinary, reproductive, musculoskeletal, lymphatic, and limitations in activities of daily living. The corresponding ICD-10 codes are listed and used in previous studies (**Supplementary Table S1**) (7). The dysfunction date was defined as the earliest recorded diagnosis date across these organ dysfunctions. Therefore, preclinical obesity was defined as confirmed excess adiposity without organ dysfunction, whereas clinical obesity was defined as confirmed excess adiposity with at least one obesity-related organ dysfunction.

### Organ dysfunction trajectory

Organ dysfunction trajectories were derived from the date of baseline measurement for two consecutive 2-year intervals. We examined two trajectory constructs, status and class change, across two periods. Period 1 began 2 years after the baseline recruitment assessment, which occurred in 2006 to 2010, and period 2 for the next consecutive 2 years. Status trajectories were defined by organ dysfunction status across the two periods: participants with no organ dysfunction at both periods were classified as no organ dysfunction; participants with organ dysfunction only in period 1; participants with organ dysfunction only in period 2; participants with organ dysfunction at both periods were classified as persistent organ dysfunction. Change trajectories were defined by the number of organ dysfunction categories across the two periods: participants with no organ dysfunction at both periods were classified as no organ dysfunction; participants with increased category numbers of organ dysfunction between period 1 and 2 as increase in organ dysfunction; participants with decreased category numbers of organ dysfunction between period 1 and 2 as decrease in organ dysfunction; participants with same number of organ dysfunction categories at both periods were classified as persistent organ dysfunction.

### Outcome

The primary outcome was incident MACE. Nonfatal events were defined from linked hospital inpatient records using ICD-10 diagnosis codes for ischemic heart disease (I21-I22) and stroke (I60-I69). Fatal cardiovascular events were identified from death registry records using the underlying cause of death coded in ICD-10 and included ischemic heart disease (I20-I25), hypertensive heart disease (I11 and I13), heart failure (I50), cerebrovascular disease (I60-I69), and other atherosclerotic and vascular causes (I70-I79), consistent with prior studies that used in previous studies (11–13). The incidence date was defined as the earliest recorded event date across hospital and death records.

### Key covariates

Selected key variables were age (continuous; years), sex (categorical; men and women), deprivation index (categorical; upper-half and lower-half), smoking status (categorical; current, former, and never smoker), alcohol consumption (categorical; yes and no), and moderate-to-vigorous physical activity (MVPA) (categorical; 0, 1-2, 3-4, ≥5 times per week). For deprivation index, we used Townsend deprivation index and by its median index, we classified into upper and lower halves. Other definitions of key variables were used in previous studies (14–16).

### Statistical analysis

We analyzed organ dysfunction trajectories by status and class change with MACE using BMI-based and clinically defined obesity. Categorical variables are presented as percentage (%) and continuous variables are represented as mean with standard deviation. For each trajectory, we calculated incidence rates (IR) per 1,000 person-years (PY) with 95% confidence interval (CI). Participants were followed from the index date at period 2 until the first occurrence of incident MACE, death, 10 years after the index date, or the end of follow up, whichever came first. We estimated adjusted hazard ratio (aHR) and 95% confidence interval (CI) for any incidence of MACE according to trajectories using Cox proportional hazards model. Model 1 was adjusted for age and sex, and Model 2 was fully adjusted for key covariates, including age, sex, deprivation index, smoking status, alcohol consumption, and MVPA. To compare the relative prognostic information provided by BMI-based and clinically defined obesity, we additionally fitted parallel Cox models in the same analytic sample with identical covariate adjustment. Comparative model performance was assessed using Harrell’s C-statistic, log-likelihood, Akaike information criterion (AIC), and likelihood-ratio tests for nested models. Subgroup analyses were performed according to sex, age, and CCI. For sensitivity analyses, we excluded events and person-time occurring within the first year of follow-up. In addition, we estimated cumulative incidence for each trajectory only while the number at risk remained ≥200. *P* value under 0.05 was considered as a statistically significant result. All analyses were conducted using SAS 9.4 (SAS Institute, Cary, NC) and RStudio version 7.2 (RStudio, New York, USA).

## Results

### Participant characteristics

This study included 457,675 UK Biobank cohort participants (men; n=251,399 [54.9%]) as eligible participants with mean age of 56.8 (8.1). 32.0% of participants were with no obesity, 57.3% with preclinical obesity, and 10.6% with clinical obesity. Participants with no obesity were more often men, had lower BMI, had lower comorbidities, and higher levels alcohol consumption. Conversely, participants with clinical obesity were more often women, had higher BMI, had higher comorbidities, and lower levels of alcohol consumption. Other baseline characteristics are presented in **Table 1**. Proportion of missingness for each variable is reported in **Supplementary Table S2**.

**Table 1:** Baseline characteristics of study population.

Characteristic	Total	No obesity	Preclinical obesity	Clinical obesity
	(n=457,675)	(n=146,665)	(n=262,422)	(n=48,588)
Age, years	56.8 (8.1)	54.9 (8.3)	57.1 (7.9)	60.7 (6.9)
Sex, n (%)				
Men	251,399 (54.9)	91,611 (62.5)	135,961 (51.8)	23,827 (49.0)
Women	206,276 (45.1)	55.054 (37.5)	126,461 (48.2)	24,761 (51.0)
BMI, kg/m^2^	27.3 (4.7)	23.3 (2.3)	29.0 (4.2)	30.6 (5.0)
Hypertension, n (%)	259,657 (56.7)	59,221 (40.4)	160,280 (61.1)	40,156 (82.7)
Diabetes, n (%)	22,369 (4.9)	2,702 (1.8)	11,989 (4.6)	7,678 (15.8)
Dyslipidemia, n (%)	202,226 (44.2)	47,808 (32.6)	123,538 (47.1)	30,880 (63.6)
Charlson comorbidity index, n (%)				
0	403,511 (88.2)	134,734 (91.9)	241,095 (91.9)	27,682 (57.0)
1	23,200 (5.1)	4,960 (3,4)	9,668 (3.7)	8,472 (17.6)
≥2	30,964 (6.8)	6,971 (4.8)	11,659 (4.4)	12,334 (25.4)
Deprivation index, n (%)				
Upper half	224,830 (49.1)	68,591 (46.8)	129,756 (49.5)	26,483 (54.5)
Lower half	232,845 (50.9)	78,074 (53.2)	132,666 (50.6)	22,105 (45.5)
Cigarette smoking, n (%)				
Current smoker	46,034 (10.1)	15,013 (10.2)	26,446 (10.1)	4,575 (9.4)
Former smoker	157,307 (34.4)	42,373 (28.9)	94,496 (35.0)	20,438 (42.1)
Never smoker	254,334 (55.6)	89,279 (60.9)	141,480 (53.9)	23,575 (48.5)
Alcohol consumption, n (%)				
Yes	372,393 (81.4)	123,096 (83.9)	213,702 (81.4)	35,595 (73.3)
No	85,282 (18.6)	23,569 (16.1)	48,720 (18.6)	12,993 (26.7)
Moderate-to-vigorous physical activity, n (%)				
Physically inactive	51,417 (11.2)	11,136 (7.6)	32,458 (12.4)	7,823 (16.1)
1-2 times of MVPA/week	67,221 (14.7)	18,076 (12.3)	41,903 (16.0)	7,242 (14.9)
3-4 times of MVPA/week	83,869 (18.3)	25,520 (17.4)	49,828 (19.0)	8,521 (17.5)
≥5 times of MVPA/week	255,168 (55.8)	91,933 (62.7)	138,233 (52.7)	24,002 (51.5)

Continuous variables were presented as mean (standard deviation) and categorical variables as n (%) unless otherwise specified. Deprivation indexes are classified into upper and lower halves by median index of Townsend deprivation index. Clinical obesity was defined as confirmed excess adiposity accompanied by objective evidence of obesity-related organ dysfunctions. Preclinicalobesity was defined as confirmed excess adiposity with preserved organ function. Obesity-related organ dysfunction was defined using ICD-10 diagnosis codes. MVPA, moderate-to-vigorous physical activity; BMI, body mass index; ICD-10, International Statistical Classification of Diseases and Related Health Problems 10th Revision.

### Comparison between BMI-based and clinically defined obesity

To directly compare BMI-based and clinically defined obesity using the same analytic sample and covariate structure, we fitted parallel Cox models. Compared with the base model, adding BMI category modestly improved prognostic discrimination and model fit, whereas adding the clinical obesity classification produced a larger improvement. The combined model including both BMI category and clinical obesity showed the best overall performance (**Supplementary Table S3**).

Likelihood-ratio testing showed that BMI category improved model fit beyond the base model, whereas the clinical obesity classification provided a substantially larger improvement. In addition, adding clinical obesity to the BMI model markedly improved model fit, whereas adding BMI category to the clinical obesity model yielded a smaller, although still statistically significant, incremental improvement (**Supplementary Table S4**). In category-specific models using the same adjustment set, compared with normal weight, underweight, overweight, and obesity were associated with adjusted hazard ratios of 1.50 (95% CI 1.29–1.73), 1.15 (95% CI 1.12–1.19), and 1.41 (95% CI 1.36–1.46), respectively. Compared with no obesity, preclinical obesity and clinical obesity were associated with adjusted hazard ratios of 1.19 (95% CI 1.16–1.23) and 1.91 (95% CI 1.84–1.99), respectively (**Supplementary Figure S1**). Calibration plots for the BMI and clinical obesity models are shown in **Supplementary Figures S2**, **S3**.

### Association between organ dysfunction trajectories and MACE risk

Across status trajectories, we observed a significant, graded increase in risk from no organ dysfunction to persistent organ dysfunction. This pattern was evident under both BMI-based and clinical-obesity classifications. Notably, the risk difference between no organ dysfunction and persistent organ dysfunction was largest among participants in the normal group [persistent organ dysfunction vs no organ dysfunction: BMI-based (aHR 3.72, 95% CI 3.20-4.33; *P* value <.001); clinically defined (aHR 3.33, 95% CI 2.73-4.06; *P* value <.001)] whereas the magnitude of association was attenuated in the obese group [persistent organ dysfunction vs no organ dysfunction: BMI-based (aHR 2.52, 95% CI 2.31-2.75; *P* value <.001); clinically defined (aHR 2.47, 95% CI 2.24-2.69; *P* value <.001)]. In addition, organ dysfunction only in period 1, compared with only in period 2, was higher in most of the trajectories (**Table 2**).

**Table 2:** Incidence rates and hazard ratios for MACE according to organ dysfunction trajectories.

Organ dysfunction trajectory^a^	Event/total	PY	IR per 1000 PY (95% CI)	Model 1^d^ (95% CI)	P for trend	Model 2^e^ (95% CI)	P for trend
BMI-based obesity^b^							
Underweight					<.001		0.002
No organ dysfunction	157/2727	23552	6.67 (5.62–7.71)	1.00 (Ref)		1.00 (Ref)	
Organ dysfunction only in period 1	20/170	1284	15.57 (8.74–22.39)	1.58 (1.11-2.27)^f^		1.48 (1.03-2.12)^g^	
Organ dysfunction only in period 2	18/146	1196	15.04 (8.09–21.99)	1.45 (0.99-2.12)		1.44 (0.99-2.10)	
Persistent organ dysfunction	8/23	163	49.03 (15.05–83.01)	4.01 (2.43-6.60)^h^		3.11 (1.85-5.20)^h^	
Normal					<.001		<.001
No organ dysfunction	5286/140494	1249153	4.23 (4.12–4.35)	1.00 (Ref)		1.00 (Ref)	
Organ dysfunction only in period 1	529/5890	49462	10.69 (9.78–11.61)	2.06 (1.92-2.21)^h^		1.97 (1.84-2.11)^h^	
Organ dysfunction only in period 2	426/5060	43247	9.85 (8.91–10.79)	1.76 (1.63-1.91)^h^		1.69 (1.56-1.83)h	
Persistent organ dysfunction	77/514	4038	19.07 (14.81–23.33)	4.01 (3.44-4.66)^h^		3.72 (3.20-4.33)^h^	
Overweight					<.001		<.001
No organ dysfunction	8742/170501	1502714	5.82 (5.70–5.94)	1.00 (Ref)		1.00 (Ref)	
Organ dysfunction only in period 1	1182/12139	102278	11.56 (10.90–12.22)	1.61 (1.53-1.69)^h^		1.55 (1.48-1.64)^h^	
Organ dysfunction only in period 2	979/10501	89835	10.90 (10.22–11.58)	1.60 (1.52-1.69)^h^		1.56 (1.48-1.64)h	
Persistent organ dysfunction	235/1345	10788	21.78 (19.00–24.57)	3.15 (2.85-3.47)^h^		2.93 (2.66-3.23)^h^	
Obese					<.001		<.001
No organ dysfunction	5169/86652	759269	6.81 (6.62–6.99)	1.00 (Ref)		1.00 (Ref)	
Organ dysfunction only in period 1	1086/10440	87595	12.40 (11.66–13.14)	1.68 (1.59-1.77)^h^		1.61 (1.53-1.70)^h^	
Organ dysfunction only in period 2	945/9278	78536	12.03 (11.27–12.80)	1.51 (1.43-1.60)^h^		1.46 (1.38-1.54)^h^	
Persistent organ dysfunction	275/1795	14472	19.00 (16.76–21.25)	2.72 (2.50-2.97)^h^		2.52 (2.31-2.75)^h^	
Clinically defined obesity^c^							
No obesity					<.001		<.001
No organ dysfunction	4536/136498	1217935	3.72 (3.62–3.83)	1.00 (Ref)		1.00 (Ref)	
Organ dysfunction only in period 1	420/5273	44489	9.44 (8.54–10.34)	1.99 (1.84-2.16)^h^		1.95 (1.79-2.11)^h^	
Organ dysfunction only in period 2	301/4466	38617	7.79 (6.91–8.67)	1.65 (1.51-1.82)^h^		1.60 (1.46-1.76)^h^	
Persistent organ dysfunction	55/428	3417	16.09 (11.84–20.35)	3.39 (2.78-4.12)^h^		3.33 (2.73-4.06)^h^	
Preclinical obesity					<.001		<.001
No organ dysfunction	11035/227042	2002586	5.51 (5.41–5.61)	1.00 (Ref)		1.00 (Ref)	
Organ dysfunction only in period 1	1556/17825	151265	10.29 (9.78–10.80)	1.65 (1.58-1.72)^h^		1.59 (1.52-1.66)^h^	
Organ dysfunction only in period 2	1371/15457	132363	10.36 (9.81–10.91)	1.63 (1.57-1.70)^h^		1.58 (1.51-1.65)^h^	
Persistent organ dysfunction	293/2098	17159	17.07 (15.12–19.03)	2.88 (2.65-3.14)^h^		2.71 (2.49-2.95)^h^	
Clinical obesity					<.001		<.001
No organ dysfunction	3783/36834	314168	12.04(11.66–12.43)	1.00 (Ref)		1.00 (Ref)	
Organ dysfunction only in period 1	841/5541	44866	18.74(17.48–20.01)	1.60 (1.51-1.70)^h^		1.53 (1.44-1.62)^h^	
Organ dysfunction only in period 2	696/5062	41834	16.64(15.40–17.87)	1.41 (1.32-1.50)^h^		1.34 (1.25-1.42)^h^	
Persistent organ dysfunction	247/1151	8883	27.80(24.34–31.27)	2.76 (2.52-3.02)^h^		2.47 (2.24-2.69)^h^	

^a^ Organ dysfunction trajectories were constructed from ICD-10 codes across 12 categories defined by the *Lancet Diabetes & Endocrinology* Commission and assessed over two consecutive 2-year intervals. Period 1 began 2 years after the baseline assessment at recruitment (2006–2010), and period 2 for the next consecutive 2 years. Status trajectories were defined by organ dysfunction status across the two periods: participants with no organ dysfunction at both periods were classified as no organ dysfunction; participants with organ dysfunction only in period 1; participants with organ dysfunction only in period 2; participants with organ dysfunction at both periods were classified as persistent organ dysfunction.

^b^ BMI-based obesity was defined by BMI. Among non-Asian participants, BMI was classified as underweight (<18.5 kg/m²), normal weight (18.5–24.9 kg/m²), overweight (25.0–29.9 kg/m²), and obese (≥30.0 kg/m²). Among Asian participants, BMI was classified as underweight (<18.5 kg/m²), normal weight (18.5–22.9 kg/m²), overweight (23.0–24.9 kg/m²), and obese (≥25.0 kg/m²).

^c^ Clinically defined obesity was defined as confirmed excess adiposity accompanied by objective evidence of obesity-related organ dysfunctions. For excess obesity, it was defined using a multi-measure anthropometric definition by BMI, WC, WHR, WHtR, and BF. Central adiposity were defined as WC ≥102 cm (men) or ≥88 cm (women) for non-Asian participants and ≥90 cm (men) or ≥80 cm (women) for Asian participants; WHR ≥0.90 (men) or ≥0.85 (women); and WHtR ≥0.50. Elevated BMI was defined as BMI ≥30 kg/m² for non-Asian participants and ≥25 kg/m² for Asian participants. Elevated BF was defined as ≥25% for men and ≥35% for women. Excess adiposity was confirmed if any of the following criteria were met: (1) elevated BF; (2) elevated BMI plus ≥1 elevated central adiposity (WC, WHR, or WHtR); or (3) ≥2 elevated central adiposity. Obesity-related organ dysfunction was defined using ICD-10 diagnosis codes.

^d^ Model 1 adjusted HR by age and sex using Cox regression.

^e^ Model 2 adjusted hazard ratios by age, sex, deprivation index, smoking status, alcohol consumption, MVPA using Cox regression.

^f^ *p* < 0.05.

^g^ *p* < 0.01.

^h^ *p* <.001.

WC, waist circumference; WHR,waist-to-hip ratio; WHtR, waist-to-height ratio; BF, body fat percentage; MVPA, moderate-to-vigorous physical activity; BMI, body mass index; ICD-10, International Statistical Classification of Diseases and Related Health Problems 10th Revision.

Similarly, change trajectories also showed a significant, graded increase in risk from no organ dysfunction to persistent organ dysfunction. The risk difference between no organ dysfunction and persistent organ dysfunction was largest among participants in the overweight group for BMI-based obesity (aHR 2.45; 95% CI 2.08-2.89; *P* value <.001) and in the normal group for clinically defined obesity (aHR 2.57; 95% CI 1.87-3.52; *P* value <.001). In addition, increase in organ dysfunction, compared with decrease, was higher in most of the trajectories (**Supplementary Tables S5, 6**).

When risk was compared across BMI-based and clinically defined obesity within strata by status trajectories, clinical obesity consistently showed the highest risk group under the clinically defined obesity [clinical obesity vs no obesity: no organ dysfunction (aHR 2.03, 95% CI 1.96-2.11; *P* value <.001); persistent organ dysfunction (aHR 1.61, 95% CI 1.30-2.01; *P* value <.001)], whereas underweight showed the highest risk group under the BMI-based obesity [underweight vs normal: no organ dysfunction (aHR 1.83, 95% CI 1.62-2.07; *P* value <.001); persistent organ dysfunction (aHR 2.19, 95% CI 1.32-3.63; *P* value <.001)] (**Table 3**).

**Table 3:** Incidence rates and hazard ratios for MACE according to BMI-based and clinically defined obesity in organ dysfunction status trajectory.

Organ dysfunction trajectory^a^	Event/total	PY	IR per 1000 PY (95% CI)	Model 1^d^ (95% CI)	P for trend	Model 2^e^ (95% CI)	P for trend
BMI-based obesity^b^							
No organ dysfunction					<.001		<.001
Underweight	277/2727	24153	11.47(10.12–12.82)	2.15 (1.91-2.42)^h^		1.83 (1.62-2.07)^h^	
Normal	7212/140494	1259500	5.73(5.59–5.86)	1.00 (Ref)		1.00 (Ref)	
Overweight	11852/170501	1519497	7.80(7.66–7.94)	1.11 (1.08-1.15)^h^		1.11 (1.08-1.14)^h^	
Obesity	7501/86652	772132	9.71(9.49–9.93)	1.51 (1.46-1.56)^h^		1.42 (1.37-1.47)^h^	
Organ dysfunction only in period 1					<.001		0.003
Underweight	34/170	1327	25.62(17.01–34.23)	1.61 (1.15-2.27)^g^		1.35 (0.96-1.91)	
Normal	891/5890	51297	17.37(16.23–18.51)	1.00 (Ref)		1.00 (Ref)	
Overweight	1784/12139	105381	16.93(16.14–17.71)	0.89 (0.82-0.97)^g^		0.88 (0.81-0.95)^g^	
Obesity	1862/10440	91478	20.35(19.43–21.28)	1.20 (1.11-1.30)^h^		1.10 (1.01-1.19)^f^	
Organ dysfunction only in period 2					<.001		0.003
Underweight	30/146	1242	24.15(15.51–32.79)	1.76 (1.22-2.53)^g^		1.53 (1.06-2.20)^f^	
Normal	660/5060	44298	14.90(13.76–16.04)	1.00 (Ref)		1.00 (Ref)	
Overweight	1594/10501	92954	17.15(16.31–17.99)	1.03 (0.94-1.12)		1.01 (0.92-1.11)	
Obesity	1503/9278	81437	18.46(17.52–19.39)	1.25 (1.14-1.37)^h^		1.15 (1.05-1.27)^g^	
Persistent organ dysfunction					0.22		0.03
Underweight	17/23	194	87.57(45.94–129.20)	2.31 (1.40-3.80)^g^		2.19 (1.32-3.63)^g^	
Normal	173/514	4391	39.39(33.52–45.26)	1.00 (Ref)		1.00 (Ref)	
Overweight	419/1345	11668	35.91(32.47–39.35)	0.86 (0.72-1.02)		0.83 (0.70-0.99)^f^	
Obesity	555/1795	15766	35.20(32.27–38.13)	0.93 (0.78-1.10)		0.85 (0.72-1.02)	
Clinically defined obesity^c^							
No organ dysfunction					<.001		<.001
No obesity	5979/136498	1225674	4.88 (4.75–5.00)	1.00 (Ref)		1.00 (Ref)	
Preclinical obesity	14875/227042	2023961	7.35 (7.23–7.47)	1.20 (1.17-1.23)^h^		1.16 (1.12-1.19)^h^	
Clinical obesity	5988/36834	325648	18.39 (17.92–18.85)	2.21 (2.13-2.29)^h^		2.03 (1.96-2.11)^h^	
Organ dysfunction only in period 1					<.001		<.001
No obesity	657/5273	45597	14.41 (13.31–15.51)	1.00 (Ref)		1.00 (Ref)	
Preclinical obesity	2433/17825	156119	15.58 (14.96–16.20)	0.99 (0.91-1.08)^h^		0.93 (0.85-1.01)	
Clinical obesity	1481/5541	47767	31.00 (29.43–32.58)	1.80 (1.64-1.98)^h^		1.58 (1.44-1.74)^h^	
Organ dysfunction only in period 2					<.001		<.001
No obesity	469/4466	39335	11.92 (10.84–13.00)	1.00 (Ref)		1.00 (Ref)	
Preclinical obesity	2127/15457	136361	15.60 (14.94–16.26)	1.17 (1.06-1.29)^g^		1.10 (1.00-1.22)	
Clinical obesity	1191/5062	44235	26.92 (25.39–28.45)	1.88 (1.69-2.10)^h^		1.67 (1.50-1.86)^h^	
Persistent organ dysfunction					<.001		<.001
No obesity	100/428	3582	27.91 (22.44–33.38)	1.00 (Ref)		1.00 (Ref)	
Preclinical obesity	546/2098	18377	29.71 (27.22–32.20)	0.99 (0.80-1.23)		0.93 (0.75-1.16)	
Clinical obesity	518/1151	10060	51.49 (47.05–55.92)	1.77 (1.43-2.19)^h^		1.61 (1.30-2.01)^h^	

^a^ Organ dysfunction trajectories were constructed from ICD-10 codes across 12 categories defined by the *Lancet Diabetes & Endocrinology* Commission and assessed over two consecutive 2-year intervals. Period 1 began 2 years after the baseline assessment at recruitment (2006–2010), and period 2 for the next consecutive 2 years. Status trajectories were defined by organ dysfunction status across the two periods: participants with no organ dysfunction at both periods were classified as no organ dysfunction; participants with organ dysfunction only in period 1; participants with organ dysfunction only in period 2; participants with organ dysfunction at both periods were classified as persistent organ dysfunction.

^b^ BMI-based obesity was defined by BMI. Among non-Asian participants, BMI was classified as underweight (<18.5 kg/m²), normal weight (18.5–24.9 kg/m²), overweight (25.0–29.9 kg/m²), and obese (≥30.0 kg/m²). Among Asian participants, BMI was classified as underweight (<18.5 kg/m²), normal weight (18.5–22.9 kg/m²), overweight (23.0–24.9 kg/m²), and obese (≥25.0 kg/m²).

^c^ Clinically defined obesity was defined as confirmed excess adiposity accompanied by objective evidence of obesity-related organ dysfunctions. For excess obesity, it was defined using a multi-measure anthropometric definition by BMI, WC, WHR, WHtR, and BF. Central adiposity were defined as WC ≥102 cm (men) or ≥88 cm (women) for non-Asian participants and ≥90 cm (men) or ≥80 cm (women) for Asian participants; WHR ≥0.90 (men) or ≥0.85 (women); and WHtR ≥0.50. Elevated BMI was defined as BMI ≥30 kg/m² for non-Asian participants and ≥25 kg/m² for Asian participants. Elevated BF was defined as ≥25% for men and ≥35% for women. Excess adiposity was confirmed if any of the following criteria were met: (1) elevated BF; (2) elevated BMI plus ≥1 elevated central adiposity (WC, WHR, or WHtR); or (3) ≥2 elevated central adiposity. Obesity-related organ dysfunction was defined using ICD-10 diagnosis codes.

^d^ Model 1 adjusted HR by age and sex using Cox regression.

^e^ Model 2 adjusted hazard ratios by age, sex, deprivation index, smoking status, alcohol consumption, MVPA using Cox regression.

^f^ *p* < 0.05.

^g^ *p* < 0.01.

^h^ *p* <.001.

WC, waist circumference; WHR,waist-to-hip ratio; WHtR, waist-to-height ratio; BF, body fat percentage; MVPA, moderate-to-vigorous physical activity; BMI, body mass index; ICD-10, International Statistical Classification of Diseases and Related Health Problems 10th Revision.

**Figure 2:**
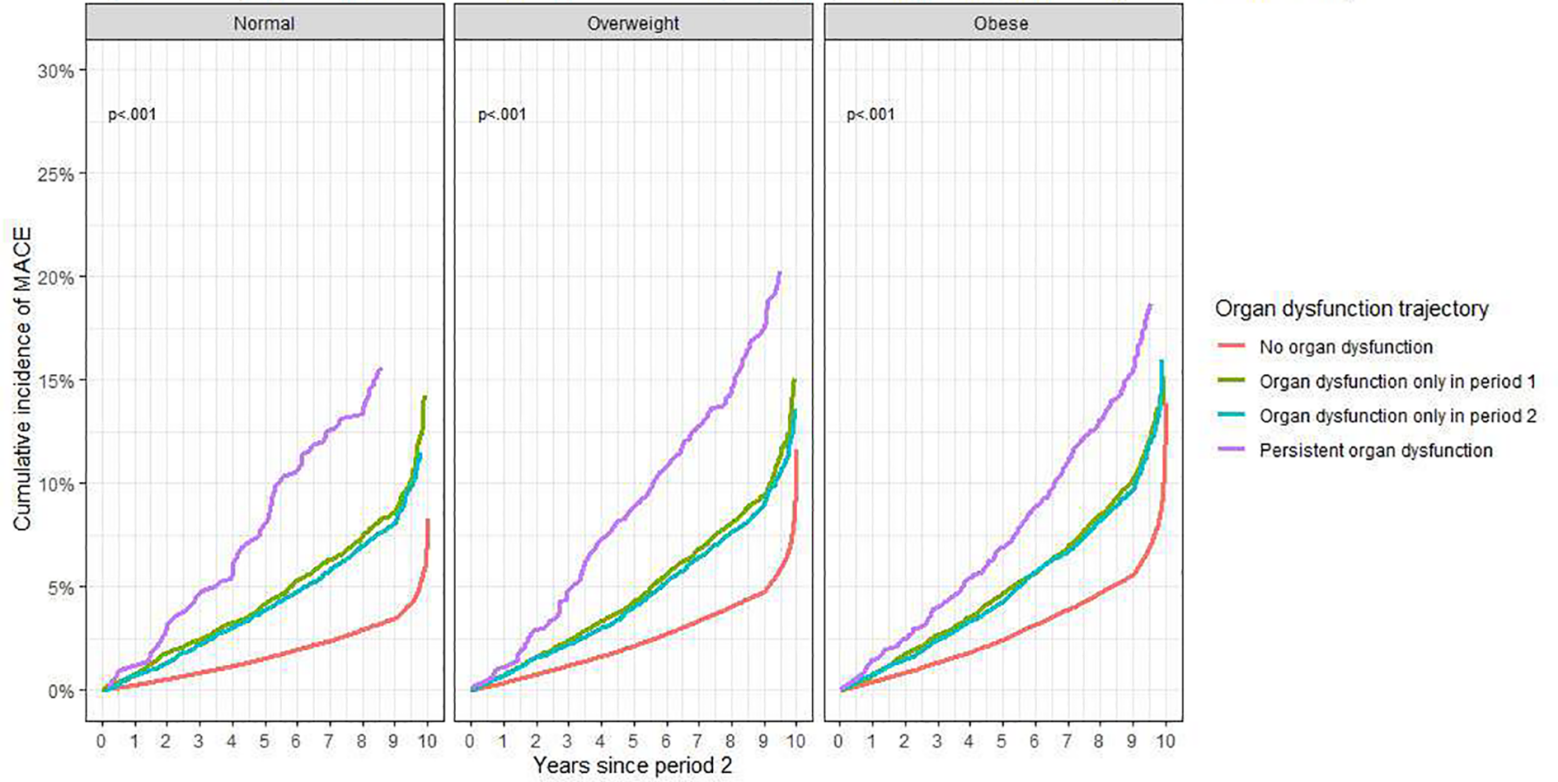
10-year cumulative incidence of major adverse cardiovascular events according to organ dysfunction status trajectories within BMI-based obesity categories. Organ dysfunction trajectories were defined across two consecutive 2-year intervals after baseline assessment. Period 1 began 2 years after the baseline recruitment assessment, and period 2 covered the subsequent 2 years. Status trajectories were classified as no organ dysfunction, organ dysfunction only in period 1, organ dysfunction only in period 2, and persistent organ dysfunction. BMI-based obesity categories were defined using ethnicity-specific body mass index cutoffs. Each panel represents a BMI-based obesity category, and curves show the cumulative incidence of major adverse cardiovascular events according to organ dysfunction trajectory. MACE, major adverse cardiovascular events; BMI, body mass index.

**Figure 3:**
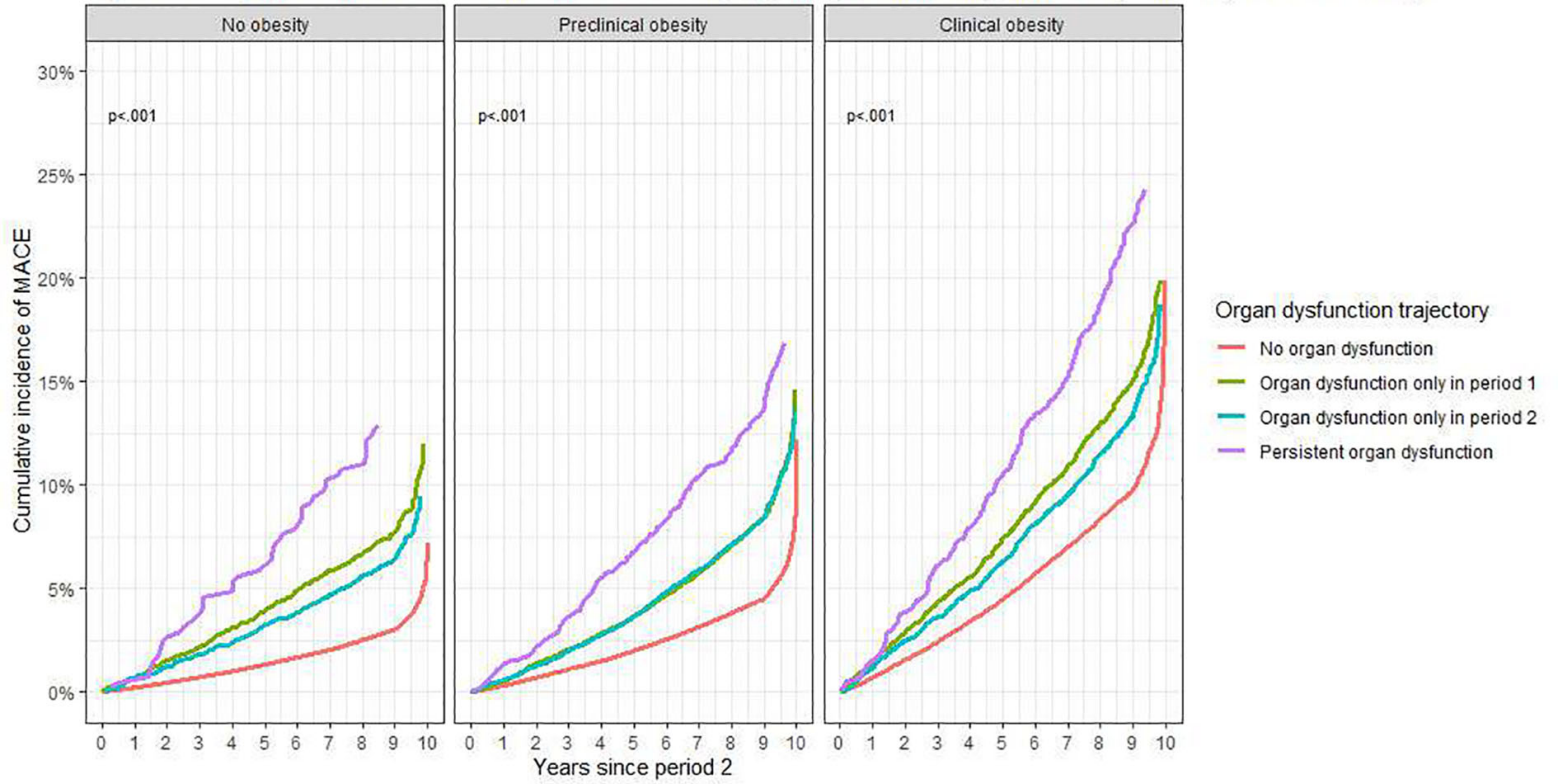
10-year cumulative incidence of major adverse cardiovascular events according to organ dysfunction status trajectories within clinically defined obesity categories. Organ dysfunction trajectories were defined across two consecutive 2-year intervals after baseline assessment. Period 1 began 2 years after the baseline recruitment assessment, and period 2 covered the subsequent 2 years. Status trajectories were classified as no organ dysfunction, organ dysfunction only in period 1, organ dysfunction only in period 2, and persistent organ dysfunction. Clinically defined obesity was classified as no obesity, preclinical obesity, or clinical obesity based on confirmed excess adiposity and obesity-related organ dysfunction. Each panel represents a clinically defined obesity category, and curves show the cumulative incidence of major adverse cardiovascular events according to organ dysfunction trajectory. MACE, major adverse cardiovascular events; BMI, body mass index.

The 10-year cumulative incidence of MACE was consistently highest in the persistent organ dysfunction trajectory across both BMI-based and clinical-obesity classifications. Within the persistent organ dysfunction trajectory, MACE incidence increased in a graded response across clinically defined obesity groups, whereas overweight group showed the highest incidence in BMI-based categories. The 10-year MACE incidence was highest in the BMI-based overweight group at approximately 20%, whereas under the clinically defined obesity, it was highest in the clinical obesity group at approximately 24% (**Figures 2**, **3**).

### Subgroup and sensitivity analysis

For BMI-based and clinically defined obesity, clinically defined obesity showed more consistent associations across subgroups, whereas some BMI-based associations were attenuated in participants with greater comorbidity burden. In higher comorbidity, preclinical obesity was no longer significantly associated with MACE, whereas clinical obesity remained associated with higher risk. In sensitivity analyses, the main findings were unchanged (**Supplementary Tables S7, S8**).

In subgroup analyses of the organ dysfunction status trajectory, similar graded patterns were observed for ischemic heart disease and stroke under clinically defined obesity. In contrast, under BMI-based categories, obesity showed the highest risk in ischemic heart disease, while underweight showed the highest risk in stroke across trajectories (**Supplementary Tables S9–S11**). However, in change trajectory, similar graded patterns were observed for ischemic heart disease and stroke under clinically defined obesity, but there was no meaningful association in increase or decrease in organ dysfunction in each MACE components under BMI-based categories (**Supplementary Tables S12–S14**).

## Discussion

We found that organ dysfunction trajectories were strongly and consistently associated with subsequent MACE, with a clear graded pattern across both trajectories. When we applied different obesity definitions, the clinically defined obesity classification captured clearer and monotonic graded increase in MACE risk across organ dysfunction trajectories, whereas the BMI-based definition showed a paradoxical increase in risk from normal to underweight, highlighting the limitations of adiposity-only classification. In addition, participants with persistent organ dysfunction had the highest risk, and those with transient dysfunction (period 1 only or 2 only) showed intermediate risk, indicating that both the duration of dysfunction and repeated assessment over time are crucial for risk stratification.

Our findings support the growing consensus that obesity-related risk is better understood as a continuum of adiposity and downstream organ impairment, rather than as excess body size alone. The Lancet Diabetes & Endocrinology Commission explicitly distinguishes preclinical obesity from clinical obesity, which incorporates excess adiposity with objective organ dysfunction or substantial functional limitation, focusing on obesity as a chronic and systemic illness (5). In the recent study that estimated incident diabetes, cardiovascular events, and mortality risk with clinical obesity, clinical obesity carried markedly elevated longitudinal risks compared with those without obesity or organ dysfunction, while organ dysfunction even without obesity also conferred substantial cardiovascular risk, highlighting organ dysfunction itself as a powerful risk (6). Similarly, a recent study using baseline and follow-up dysfunction patterns reported the highest mortality risk among those with clinical obesity at baseline, and notably high risk among those with dysfunction even in the absence of obesity, emphasizing the central role of dysfunction status than BMI alone (7). While prior studies applying the clinically defined obesity definition have shown that obesity-related dysfunction identifies individuals at higher cardiometabolic risk, these analyses have generally classified dysfunction cross-sectionally rather than evaluating organ dysfunction over repeated assessments in trajectories.

Another key contribution of our study is that we measured dysfunction by trajectories, which is shown to better stratify risks among many epidemiology studies (17–23). Across status trajectories, participants showed graded risk and incidence increase from transient dysfunction to persistent dysfunction, suggesting that persistence and cumulative exposure to dysfunction may be more prognostic for MACE than dysfunction in a single assessment (24–26). In addition, Xu et al. used a multistate model to track progression from preclinical obesity to obesity induced dysfunctions and death, directly supporting the clinical relevance of dysfunction progression rather than single assessment (8). Persistent multi-organ dysfunction may reflect sustained inflammatory and neurohumoral dysregulation, contributing to plaque inflammation, instability, rupture, and thrombosis, providing a plausible pathway that links to sustained dysfunction burden to MACE (27). The observation that organ dysfunction only in period 1 often carried higher risk than dysfunction only in period 2 may similarly indicate longer exposure duration of underlying dysfunction before the outcome.

While BMI-based categories showed heterogeneity in risk, the clinical-obesity classification demonstrated a more monotonic, graded increase in MACE risk across obesity categories compared to BMI-based categories, especially within high-risk dysfunction trajectories. By contrast, the BMI-based definition showed higher risk in the underweight group than in the normal group. It may be due to reverse causation where low BMI is a marker of underlying illness, frailty, or sarcopenia rather than a truly protective state (4). Prior population studies have documented elevated cardiovascular and mortality risk among underweight individuals, which is attributed to concomitant comorbid conditions, chronic disease burden, or loss of muscle mass rather than the direct causal effect of low adiposity alone (3, 28). These findings suggest that clinical obesity better captures the pathophysiological burden relevant to cardiovascular events than BMI alone, which is an imperfect surrogate because it cannot differentiate fat from lean mass or capture fat distribution (29). Alternative adiposity measures, including waist-to-hip ratio, waist-to-height ratio, and visceral fat, may better reflect central or ectopic adiposity and may therefore provide additional cardiovascular risk information beyond BMI alone. Our clinically defined obesity framework partly addresses this by incorporating multiple anthropometric indicators and obesity-related organ dysfunction. In addition, BMI-based classification can confuse individuals with high BMI but relatively preserved metabolic/organ function with those who have clinically meaningful dysfunction, potentially underestimating risk (30, 31). Conversely, clinical obesity more directly reflects the presence of end-organ damage due to excess adiposity, improving risk stratification for cardiovascular outcomes.

However, the generalizability of our findings should be interpreted with caution because the UK Biobank is predominantly composed of White participants from the United Kingdom. Differences in baseline cardiovascular risk, body composition, fat distribution, and the relationship between BMI and excess adiposity across racial and ethnic groups may influence the performance of both BMI-based and clinically defined obesity frameworks. Therefore, the association may differ in populations with different adiposity distributions and metabolic profiles.

Our study has several limitations. First, we measured adiposity at baseline and could not track changes of adiposity during follow-up, which may have misclassification bias. Second, obesity-related organ dysfunction was defined using ICD-10 diagnosis codes, which can cause misclassification bias. However, ICD-10 codes have been widely used and validated in many prior epidemiologic studies (11, 18, 32–35). Third, because few studies have analyzed organ dysfunctions in trajectories, direct comparisons with existing literature are limited. Finally, trajectories were constructed over two discrete periods, which may not fully reflect longer-term fluctuations or relapse patterns over the life course. Fourth, our study was based on the UK Biobank, which predominantly includes White participants from the United Kingdom, and this may limit generalizability to more diverse populations or healthcare settings. The relationship between BMI, body fat distribution, and obesity-related organ dysfunction may differ across racial and ethnic groups with different baseline cardiovascular risk profiles. Therefore, multi-ethnic cohort validation is further needed.

In conclusion, the clinically defined obesity classification more consistently identified high-risk trajectories for MACE, showing a clearer and more monotonic graded increase in risk than BMI-based categories, which were more susceptible to paradoxical BMI patterns including elevated risk among underweight individuals. Repeated assessments of organ dysfunction by trajectories also improved risk estimation. Incorporating clinically defined obesity definitions with longitudinal assessment of organ dysfunction may therefore enhance cardiovascular risk stratification beyond BMI alone.

## Data Availability

The data analyzed in this study is subject to the following licenses/restrictions: The data used in this study are available from the UK Biobank (https://www.ukbiobank.ac.uk/enable-your-research/apply-for-access). As restrictions apply to the availability of these data, which were used under license for the current study, the authors cannot publicly share these data. This research has been conducted using the UK Biobank Resource under Application Number 106954. Requests to access these datasets should be directed to https://www.ukbiobank.ac.uk/.
