# Supplementary material for: Association of organ dysfunction trajectories and major adverse cardiovascular events using clinical obesity in UK Biobank
Source: Front Endocrinol (Lausanne). 2026 May 14;17:1844870. doi: 10.3389/fendo.2026.1844870 (PMC13215929; doi:10.3389/fendo.2026.1844870)
Supplement: Supplementary file 1 [file DataSheet1.docx]

**Supplementary Material**

**“Association of Organ Dysfunction Trajectories and Major Adverse Cardiovascular Events using Clinical Obesity in UK Biobank”**

**Table S1.** List of organ dysfunctions due to obesity or limitations of daily activities by ICD-10 codes

**Table S2.** Missingness of variables used in the analysis

**Table S3.** Model performance of BMI-based and clinically defined obesity frameworks for incident major adverse cardiovascular events

**Table S4.** Likelihood-ratio comparison of nested Cox models for BMI-based and clinically defined obesity frameworks in relation to incident major adverse cardiovascular events

**Table S5.** Hazard ratios for MACE according to organ dysfunction change trajectory in BMI-based obesity

**Table S6.** Hazard ratios for MACE according to organ dysfunction change trajectory in clinically defined obesity

**Table S7.** Subgroup and sensitivity analyses for BMI-based obesity with incident MACE

**Table S8.** Subgroup and sensitivity analyses for clinical obesity with incident MACE

**Table S9**. Hazard ratios for ischemic heart disease according to BMI-based and clinically defined obesity in status trajectory

**Table S10.** Hazard ratios for stroke according to BMI-based and clinically defined obesity in status trajectory

**Table S11.** Hazard ratios for fatal cardiovascular disease according to BMI-based and clinically defined obesity in status trajectory

**Table S12.** Hazard ratios for ischemic heart disease according to BMI-based and clinically defined obesity in change trajectory

**Table S13.** Hazard ratios for stroke according to BMI-based and clinically defined obesity in change trajectory

**Table S14.** Hazard ratios for fatal cardiovascular disease according to BMI-based and clinically defined obesity in change trajectory

**Figure S1.** Side-by-side adjusted hazard ratios for BMI-based and clinically defined obesity in relation to incident major adverse cardiovascular events.
**Figure S2.** Calibration plot for the BMI category model at 10 years.
**Figure S3.** Calibration plot for the clinical obesity model at 10 years.

**Table S1.** List of organ dysfunctions due to obesity or limitations of daily activities by ICD-10 codes

| **Organ, tissue, body system** | **ICD-10 codes** |
| --- | --- |
| Central nervous system | G93.2 + H54/R51/G43/G44 |
| Upper airway | G47 |
| Respiratory | J44; restricted to never smoker |
| Cardiovascular | (I50.2, I50.3, I48.2, I27, I26, I82, I10 |
| Metabolic | R73, E11, E78 |
| Liver | K75.81, K74.0, K76.0 |
| Renal | N18 |
| Urinary | N39.3, N39.4, R39 |
| Reproductive | female: E28.2, N91, E28; male: E29 |
| Musculoskeletal | M16, M17, M25.5, M25.65, M25.66 |
| Lymphatic | I89.0 |
| Limitations in activities of daily living | R53.1, Z74, Z73.6 |

ICD-10 codes are based on the Lancet Commission framework for definition of clinical obesity.

Acronyms: ICD-10, International Statistical Classification of Diseases and Related Health Problems 10th Revision.

**Table S2.** Missingness of variables used in the analysis

| **Variable** | **No. missing** | **% missing** |
| --- | --- | --- |
| Baseline assessment date | 0 | 0.00 |
| Baseline obesity classification unavailable | 2084 | 0.42 |
| Body mass index | 2963 | 0.60 |
| Waist circumference | 2081 | 0.42 |
| Hip circumference | 2133 | 0.43 |
| Height | 2432 | 0.49 |
| Body fat percentage | 9851 | 2.00 |
| Age at interview start | 0 | 0.00 |
| Sex | 0 | 0.00 |
| Moderate-to-vigorous physical activity group | 21115 | 4.30 |
| Alcohol consumption | 1221 | 0.25 |
| Smoking status | 2488 | 0.51 |

**Table S3.** Model performance of BMI-based and clinically defined obesity frameworks for incident major adverse cardiovascular events

| **Model** | **N** | **Events** | **Log-likelihood** | **AIC** |
| --- | --- | --- | --- | --- |
| Base | 457,675 | 23,773 | -297,812.3 | 595,642.6 |
| Base + BMI category | 457,675 | 23,773 | -297,623.1 | 595,270.2 |
| Base + Clinical obesity | 457,675 | 23,773 | -297,289.8 | 594,601.6 |
| Base + BMI + Clinical obesity | 457,675 | 23,773 | -297,240.3 | 594,508.5 |

Base model was adjusted for age, sex, moderate-to-vigorous physical activity, alcohol consumption, Townsend deprivation index, and smoking status. BMI category was added using ethnicity-specific BMI cutoffs. Clinical obesity was classified as no obesity, preclinical obesity, or clinical obesity. C-statistic indicates Harrell’s concordance statistic from Cox proportional hazards models. Lower AIC values indicate better model fit.

**Table S4.** Likelihood-ratio comparison of nested Cox models for BMI-based and clinically defined obesity frameworks in relation to incident major adverse cardiovascular events

| **Comparison** | **LR χ²** | **df** | **P value** |
| --- | --- | --- | --- |
| Base vs Base + BMI category | 378.4 | 3 | <0.001 |
| Base vs Base + Clinical obesity | 1044.9 | 2 | <0.001 |
| Base + BMI vs Base + BMI + Clinical obesity | 765.6 | 2 | <0.001 |
| Base + Clinical obesity vs Base + BMI + Clinical obesity | 99.1 | 3 | <0.001 |

Likelihood-ratio tests compared nested Cox proportional hazards models fitted in the same analytic sample. The base model included age, sex, moderate-to-vigorous physical activity, alcohol consumption, Townsend deprivation index, and smoking status. BMI category and clinical obesity were added separately or jointly to evaluate their incremental contribution to model fit.

**Table S5.** Hazard ratios for MACE according to organ dysfunction change trajectory in BMI-based obesity

| **Organ dysfunction trajectory^a^** | **Model^c^ (95% CI)** | ***P* for trend** |
| --- | --- | --- |
| **BMI-based obesity^b^** |  |  |
| **Underweight** |  | 0.003 |
| No organ dysfunction | 1.00 (Ref) |  |
| Increase in organ dysfunction | 1.75 (1.11-2.75)^d^ |  |
| Decrease in organ dysfunction | 1.72 (1.07-2.76)^d^ |  |
| Persistent organ dysfunction | 1.99 (0.48-8.18) |  |
| **Normal** |  | <.001 |
| No organ dysfunction | 1.00 (Ref) |  |
| Increase in organ dysfunction | 1.67 (1.52-1.83)^f^ |  |
| Decrease in organ dysfunction | 1.62 (1.47-1.79)^f^ |  |
| Persistent organ dysfunction | 2.11 (1.56-2.84)^f^ |  |
| **Overweight** |  | <.001 |
| No organ dysfunction | 1.00 (Ref) |  |
| Increase in organ dysfunction | 1.50 (1.41-1.60)^f^ |  |
| Decrease in organ dysfunction | 1.41 (1.32-1.50)^f^ |  |
| Persistent organ dysfunction | 2.45 (2.08-2.89)^f^ |  |
| **Obese** |  | <.001 |
| No organ dysfunction | 1.00 (Ref) |  |
| Increase in organ dysfunction | 1.43 (1.34-1.53)^f^ |  |
| Decrease in organ dysfunction | 1.43 (1.33-1.53)^f^ |  |
| Persistent organ dysfunction | 2.01 (1.71-2.36)^f^ |  |

^a^Organ dysfunction trajectories were constructed from ICD-10 codes across 12 categories defined by the *Lancet Diabetes & Endocrinology* Commission and assessed over two consecutive 2-year intervals. Period 1 began 2 years after the baseline assessment at recruitment (2006–2010), and period 2 for the next consecutive 2 years. Change trajectories were defined by the number of organ dysfunction categories across the two periods: participants with no organ dysfunction at both periods were classified as no organ dysfunction; participants with increased category numbers of organ dysfunction between period 1 and 2 as increase in organ dysfunction; participants with decreased category numbers of organ dysfunction between period 1 and 2 as decrease in organ dysfunction; participants with same number of organ dysfunction categories at both periods were classified as persistent organ dysfunction.

^b^BMI-based obesity was defined by BMI. Participants were classified as obese if BMI was **≥**30 kg/m² for non-Asian or ≥25 kg/m**²** for Asian participants; otherwise, they were classified as non-obese.

^c^Model adjusted hazard ratios by age, sex, deprivation index, smoking status, alcohol consumption, MVPA using Cox regression..

^d^*p*<0.05

^e^*p*<0.01

^f^*p*<.001

Acronyms: WC, waist circumference; WHR,waist-to-hip ratio; WHtR, waist-to-height ratio; BF, body fat percentage; MVPA, moderate-to-vigorous physical activity; BMI, body mass index; ICD-10, International Statistical Classification of Diseases and Related Health Problems 10th Revision.

**Table S6.** Hazard ratios for MACE according to organ dysfunction change trajectory in clinically defined obesity

| **Organ dysfunction trajectory^a^** | **Model^c^ (95% CI)** | ***P* for trend** |
| --- | --- | --- |
| **Clinically defined obesity^b^** |  |  |
| **No obesity** |  | <.001 |
| No organ dysfunction | 1.00 (Ref) |  |
| Increase in organ dysfunction | 1.69 (1.53-1.88)^f^ |  |
| Decrease in organ dysfunction | 1.42 (1.26-1.60)^f^ |  |
| Persistent organ dysfunction | 2.57 (1.87-3.52)^f^ |  |
| **Preclinical obesity** |  | <.001 |
| No organ dysfunction | 1.00 (Ref) |  |
| Increase in organ dysfunction | 1.46 (1.38-1.54)^f^ |  |
| Decrease in organ dysfunction | 1.49 (1.41-1.58)^f^ |  |
| Persistent organ dysfunction | 1.96 (1.67-2.30)^f^ |  |
| **Clinical obesity** |  | <.001 |
| No organ dysfunction | 1.00 (Ref) |  |
| Increase in organ dysfunction | 1.43 (1.33-1.54)^f^ |  |
| Decrease in organ dysfunction | 1.30 (1.20-1.41)^f^ |  |
| Persistent organ dysfunction | 2.04 (1.74-2.40)^f^ |  |

^a^Organ dysfunction trajectories were constructed from ICD-10 codes across 12 categories defined by the *Lancet Diabetes & Endocrinology* Commission and assessed over two consecutive 2-year intervals. Period 1 began 2 years after the baseline assessment at recruitment (2006–2010), and period 2 for the next consecutive 2 years. Change trajectories were defined by the number of organ dysfunction categories across the two periods: participants with no organ dysfunction at both periods were classified as no organ dysfunction; participants with increased category numbers of organ dysfunction between period 1 and 2 as increase in organ dysfunction; participants with decreased category numbers of organ dysfunction between period 1 and 2 as decrease in organ dysfunction; participants with same number of organ dysfunction categories at both periods were classified as persistent organ dysfunction.

^b^Clinical obesity was defined as confirmed excess adiposity accompanied by objective evidence of obesity-related organ dysfunctions. For excess obesity, it was defined using a multi-measure anthropometric definition by BMI, WC, WHR, WHtR, and BF. Central adiposity were defined as WC ≥102 cm (men) or ≥88 cm (women) for non-Asian participants and ≥90 cm (men) or ≥80 cm (women) for Asian participants; WHR ≥0.90 (men) or ≥0.85 (women); and WHtR ≥0.50. Elevated BMI was defined as BMI ≥30 kg/m² for non-Asian participants and ≥25 kg/m² for Asian participants. Elevated BF was defined as ≥25% for men and ≥35% for women. Excess adiposity was confirmed if any of the following criteria were met: (1) elevated BF; (2) elevated BMI plus ≥1 elevated central adiposity (WC, WHR, or WHtR); or (3) ≥2 elevated central adiposity. Obesity-related organ dysfunction was defined using ICD-10 diagnosis codes.

^c^Model adjusted hazard ratios by age, sex, deprivation index, smoking status, alcohol consumption, MVPA using Cox regression.

^d^*p*<0.05

^e^*p*<0.01

^f^*p*<.001

Acronyms: CI, confidence interval; WC, waist circumference; WHR,waist-to-hip ratio; WHtR, waist-to-height ratio; BF, body fat percentage; MVPA, moderate-to-vigorous physical activity; BMI, body mass index; ICD-10, International Statistical Classification of Diseases and Related Health Problems 10th Revision.

**Table S7.** Subgroup and sensitivity analyses for BMI-based obesity with incident MACE

| **Subgroup** | **Underweight vs normal** | **Overweight vs normal** | **Obese vs normal** | **P for interaction** |
| --- | --- | --- | --- | --- |
| **Sex** |  |  |  | **0.066** |
| Women | 1.42 (1.17–1.71)^c^ | 1.17 (1.12–1.23)^c^ | 1.42 (1.35–1.50)^c^ |  |
| Men | 1.71 (1.39–2.09)^c^ | 1.11 (1.07–1.16)^c^ | 1.40 (1.33–1.46)^c^ |  |
| **Age group** |  |  |  | **<.001** |
| <60 years | 1.78 (1.41–2.23)^c^ | 1.24 (1.17–1.31)^c^ | 1.63 (1.54–1.73)^c^ |  |
| ≥60 years | 1.43 (1.20–1.71)^c^ | 1.10 (1.06–1.14)^c^ | 1.33 (1.27–1.38)^c^ |  |
| **Charlson Comorbidity Index** |  |  |  | **<.001** |
| 0 | 1.25 (1.04–1.50)^a^ | 1.15 (1.11–1.19)^c^ | 1.37 (1.32–1.43)^c^ |  |
| 1 | 1.92 (1.37–2.69)^c^ | 1.05 (0.94–1.17) | 1.17 (1.04–1.31)^b^ |  |
| ≥2 | 1.75 (1.31–2.34)^c^ | 1.07 (0.98–1.17) | 1.36 (1.24–1.49)^c^ |  |
| **Sensitivity analysis** |  |  |  |  |
| Excluding events within the first year of follow-up | 1.55 (1.34–1.79)^c^ | 1.14 (1.11–1.18)^c^ | 1.42 (1.37–1.47)^c^ |  |

Adjusted hazard ratios (95% confidence intervals) from Cox proportional hazards models. Subgroup models were adjusted for age at interview start, sex, moderate-to-vigorous physical activity, alcohol consumption, Townsend deprivation index, and smoking status; when sex was the stratifying variable, sex was omitted from adjustment. Age group was defined as <60 and ≥60 years. Charlson Comorbidity Index was categorized as 0, 1, and ≥2. P for interaction was derived from multiplicative interaction terms between BMI category and the corresponding subgroup variable in the fully adjusted model. Sensitivity analysis excluded MACE events occurring within the first year of follow-up.

^a^*p*<0.05

^b^*p*<0.01

^c^*p*<.001

**Table S8.** Subgroup and sensitivity analyses for clinical obesity with incident MACE

| **Subgroup** | **Preclinical obesity vs no obesity** | **Clinical obesity vs no obesity** | **P for interaction** |
| --- | --- | --- | --- |
| **Sex** |  |  | **<.001** |
| Women | 1.13 (1.08–1.19)^c^ | 1.96 (1.85–2.08)^c^ |  |
| Men | 1.23 (1.18–1.29)^c^ | 1.97 (1.87–2.07)^c^ |  |
| **Age group** |  |  | **<.001** |
| <60 years | 1.33 (1.26–1.41)^c^ | 2.51 (2.33–2.70)^c^ |  |
| ≥60 years | 1.12 (1.07–1.16)^c^ | 1.77 (1.69–1.85)^c^ |  |
| **Charlson Comorbidity Index** |  |  | **<.001** |
| 0 | 1.25 (1.20–1.29)^c^ | 1.60 (1.52–1.68)^c^ |  |
| 1 | 0.99 (0.87–1.12) | 1.23 (1.09–1.39)^c^ |  |
| ≥2 | 0.98 (0.88–1.09) | 1.76 (1.59–1.93)^c^ |  |
| **Sensitivity analysis** |  |  |  |
| Excluding events within the first year of follow-up | 1.20 (1.16–1.24)^c^ | 1.95 (1.87–2.03)^c^ |  |

Adjusted hazard ratios (95% confidence intervals) from Cox proportional hazards models. Subgroup models were adjusted for age, sex, moderate-to-vigorous physical activity, alcohol consumption, Townsend deprivation index, and smoking status; when sex was the stratifying variable, sex was omitted from adjustment. Age group was defined as <60 and ≥60 years. Charlson Comorbidity Index was categorized as 0, 1, and ≥2. P for interaction was derived from multiplicative interaction terms between clinical obesity category and the corresponding subgroup variable in the fully adjusted model. Sensitivity analysis excluded MACE events occurring within the first year of follow-up.

^a^*p*<0.05

^b^*p*<0.01

^c^*p*<.001

**Table S9.** Hazard ratios for ischemic heart disease according to BMI-based and clinically defined obesity in status trajectory

| **Organ dysfunction trajectory^a^** | **Event/Total** | **Model^d^ (95% CI)** | ***P* for trend** |
| --- | --- | --- | --- |
| **BMI-based obesity^b^** |  |  |  |
| **No organ dysfunction** |  |  | <.001 |
| Underweight | 41/2727 | 1.41 (1.10-1.81)^f^ |  |
| Normal | 1720/140494 | 1.00 (Ref) |  |
| Overweight | 3398/170501 | 1.27 (1.21-1.34)^g^ |  |
| Obesity | 2078/86652 | 1.74 (1.64-1.84)^g^ |  |
| **Organ dysfunction only in period 1** |  |  | 0.53 |
| Underweight | 5/170 | 0.66 (0.27-1.60) |  |
| Normal | 146/5890 | 1.00 (Ref) |  |
| Overweight | 405/12139 | 0.97 (0.84-1.13) |  |
| Obesity | 328/10440 | 1.02 (0.88-1.19) |  |
| **Organ dysfunction only in period 2** |  |  | <.001 |
| Underweight | 3/146 | 0.82 (0.34-2.00) |  |
| Normal | 122/5060 | 1.00 (Ref) |  |
| Overweight | 311/10501 | 0.97 (0.82-1.15) |  |
| Obesity | 331/9278 | 1.26 (1.06-1.49)^f^ |  |
| **Persistent organ dysfunction** |  |  | 0.78 |
| Underweight | 4/23 | 4.32 (2.01-9.25)^g^ |  |
| Normal | 17/514 | 1.00 (Ref) |  |
| Overweight | 81/1345 | 0.88 (0.63-1.24) |  |
| Obesity | 103/1795 | 1.05 (0.76-1.47) |  |
| **Clinically defined obesity^c^** |  |  |  |
| **No organ dysfunction** |  |  | <.001 |
| No obesity | 1514/136498 | 1.00 (Ref) |  |
| Preclinical obesity | 4388/227042 | 1.41 (1.33-1.49)^g^ |  |
| Clinical obesity | 1335/36834 | 2.39 (2.24-2.55)^g^ |  |
| **Organ dysfunction only in period 1** |  |  | <.001 |
| No obesity | 116/5273 | 1.00 (Ref) |  |
| Preclinical obesity | 481/17825 | 1.15 (0.96-1.36) |  |
| Clinical obesity | 287/5541 | 2.07 (1.73-2.49)^g^ |  |
| **Organ dysfunction only in period 2** |  |  | <.001 |
| No obesity | 80/4466 | 1.00 (Ref) |  |
| Preclinical obesity | 456/15457 | 1.33 (1.10-1.61)^f^ |  |
| Clinical obesity | 231/5062 | 1.95 (1.59-2.39)^g^ |  |
| **Persistent organ dysfunction** |  |  | <.001 |
| No obesity | 12/428 | 1.00 (Ref) |  |
| Preclinical obesity | 104/2098 | 1.45 (0.91-2.31) |  |
| Clinical obesity | 89/1151 | 3.44 (2.15-5.49)^g^ |  |

^a^Organ dysfunction trajectories were constructed from ICD-10 codes across 12 categories defined by the *Lancet Diabetes & Endocrinology* Commission and assessed over two consecutive 2-year intervals. Period 1 began 2 years after the baseline assessment at recruitment (2006–2010), and period 2 for the next consecutive 2 years. Status trajectories were defined by organ dysfunction status across the two periods: participants with no organ dysfunction at both periods were classified as no organ dysfunction; participants with organ dysfunction only in period 1; participants with organ dysfunction only in period 2; participants with organ dysfunction at both periods were classified as persistent organ dysfunction.

^b^BMI-based obesity was defined by BMI. Among non-Asian participants, BMI was classified as underweight (<18.5 kg/m²), normal weight (18.5–24.9 kg/m²), overweight (25.0–29.9 kg/m²), and obese (≥30.0 kg/m²). Among Asian participants, BMI was classified as underweight (<18.5 kg/m²), normal weight (18.5–22.9 kg/m²), overweight (23.0–24.9 kg/m²), and obese (≥25.0 kg/m²).

^c^Clinical obesity was defined as confirmed excess adiposity accompanied by objective evidence of obesity-related organ dysfunctions. For excess obesity, it was defined using a multi-measure anthropometric definition by BMI, WC, WHR, WHtR, and BF. Central adiposity were defined as WC ≥102 cm (men) or ≥88 cm (women) for non-Asian participants and ≥90 cm (men) or ≥80 cm (women) for Asian participants; WHR ≥0.90 (men) or ≥0.85 (women); and WHtR ≥0.50. Elevated BMI was defined as BMI ≥30 kg/m² for non-Asian participants and ≥25 kg/m² for Asian participants. Elevated BF was defined as ≥25% for men and ≥35% for women. Excess adiposity was confirmed if any of the following criteria were met: (1) elevated BF; (2) elevated BMI plus ≥1 elevated central adiposity (WC, WHR, or WHtR); or (3) ≥2 elevated central adiposity. Obesity-related organ dysfunction was defined using ICD-10 diagnosis codes.

^d^Model adjusted hazard ratios by age, sex, deprivation index, smoking status, alcohol consumption, MVPA using Cox regression.

^e^*p*<0.05

^f^*p*<0.01

^g^*p*<.001

Acronyms: WC, waist circumference; WHR,waist-to-hip ratio; WHtR, waist-to-height ratio; BF, body fat percentage; MVPA, moderate-to-vigorous physical activity; BMI, body mass index; ICD-10, International Statistical Classification of Diseases and Related Health Problems 10th Revision; NA, not applicable.

**Table S10.** Hazard ratios for stroke according to BMI-based and clinically defined obesity in status trajectory

| **Organ dysfunction trajectory^a^** | **Event/Total** | **Model^d^ (95% CI)** | ***P* for trend** |
| --- | --- | --- | --- |
| **BMI-based obesity^b^** |  |  |  |
| **No organ dysfunction** |  |  | <.001 |
| Underweight | 118/2727 | 2.05 (1.79-2.35)^g^ |  |
| Normal | 3608/140494 | 1.00 (Ref) |  |
| Overweight | 5418/170501 | 1.04 (1.00-1.08)^e^ |  |
| Obesity | 3117/86652 | 1.28 (1.23-1.33)^g^ |  |
| **Organ dysfunction only in period 1** |  |  | 0.002 |
| Underweight | 15/170 | 1.63 (1.12-2.37)^f^ |  |
| Normal | 387/5890 | 1.00 (Ref) |  |
| Overweight | 796/12139 | 0.86 (0.78-0.95)^f^ |  |
| Obesity | 768/10440 | 1.13 (1.03-1.24)^e^ |  |
| **Organ dysfunction only in period 2** |  |  | 0.22 |
| Underweight | 15/146 | 1.83 (1.22-2.73)^f^ |  |
| Normal | 306/5060 | 1.00 (Ref) |  |
| Overweight | 678/10501 | 1.04 (0.93-1.16) |  |
| Obesity | 620/9278 | 1.11 (0.99-1.24) |  |
| **Persistent organ dysfunction** |  |  | 0.01 |
| Underweight | 4/23 | 1.49 (0.75-2.95) |  |
| Normal | 62/514 | 1.00 (Ref) |  |
| Overweight | 156/1345 | 0.79 (0.65-0.98)^e^ |  |
| Obesity | 174/1795 | 0.78 (0.63-0.95)^e^ |  |
| **Clinically defined obesity^c^** |  |  |  |
| **No organ dysfunction** |  |  | <.001 |
| No obesity | 3066/136498 | 1.00 (Ref) |  |
| Preclinical obesity | 6719/227042 | 1.04 (1.00-1.08)^e^ |  |
| Clinical obesity | 2476/36834 | 1.87 (1.78-1.95)^g^ |  |
| **Organ dysfunction only in period 1** |  |  | <.001 |
| No obesity | 305/5273 | 1.00 (Ref) |  |
| Preclinical obesity | 1097/17825 | 0.88 (0.80-0.97)^e^ |  |
| Clinical obesity | 564/5541 | 1.45 (1.30-1.62)^g^ |  |
| **Organ dysfunction only in period 2** |  |  | <.001 |
| No obesity | 223/4466 | 1.00 (Ref) |  |
| Preclinical obesity | 923/15457 | 1.02 (0.91-1.15) |  |
| Clinical obesity | 473/5062 | 1.59 (1.40-1.80)^g^ |  |
| **Persistent organ dysfunction** |  |  | 0.002 |
| No obesity | 46/428 | 1.00 (Ref) |  |
| Preclinical obesity | 192/2098 | 0.79 (0.63-0.99)^e^ |  |
| Clinical obesity | 158/1151 | 1.15 (0.89-1.45) |  |

^a^Organ dysfunction trajectories were constructed from ICD-10 codes across 12 categories defined by the *Lancet Diabetes & Endocrinology* Commission and assessed over two consecutive 2-year intervals. Period 1 began 2 years after the baseline assessment at recruitment (2006–2010), and period 2 for the next consecutive 2 years. Status trajectories were defined by organ dysfunction status across the two periods: participants with no organ dysfunction at both periods were classified as no organ dysfunction; participants with organ dysfunction only in period 1; participants with organ dysfunction only in period 2; participants with organ dysfunction at both periods were classified as persistent organ dysfunction.

^b^BMI-based obesity was defined by BMI. Among non-Asian participants, BMI was classified as underweight (<18.5 kg/m²), normal weight (18.5–24.9 kg/m²), overweight (25.0–29.9 kg/m²), and obese (≥30.0 kg/m²). Among Asian participants, BMI was classified as underweight (<18.5 kg/m²), normal weight (18.5–22.9 kg/m²), overweight (23.0–24.9 kg/m²), and obese (≥25.0 kg/m²).

^c^Clinical obesity was defined as confirmed excess adiposity accompanied by objective evidence of obesity-related organ dysfunctions. For excess obesity, it was defined using a multi-measure anthropometric definition by BMI, WC, WHR, WHtR, and BF. Central adiposity were defined as WC ≥102 cm (men) or ≥88 cm (women) for non-Asian participants and ≥90 cm (men) or ≥80 cm (women) for Asian participants; WHR ≥0.90 (men) or ≥0.85 (women); and WHtR ≥0.50. Elevated BMI was defined as BMI ≥30 kg/m² for non-Asian participants and ≥25 kg/m² for Asian participants. Elevated BF was defined as ≥25% for men and ≥35% for women. Excess adiposity was confirmed if any of the following criteria were met: (1) elevated BF; (2) elevated BMI plus ≥1 elevated central adiposity (WC, WHR, or WHtR); or (3) ≥2 elevated central adiposity. Obesity-related organ dysfunction was defined using ICD-10 diagnosis codes.

^d^Model adjusted hazard ratios by age, sex, deprivation index, smoking status, alcohol consumption, MVPA using Cox regression.

^e^*p*<0.05

^f^*p*<0.01

^g^*p*<.001

Acronyms: WC, waist circumference; WHR,waist-to-hip ratio; WHtR, waist-to-height ratio; BF, body fat percentage; MVPA, moderate-to-vigorous physical activity; BMI, body mass index; ICD-10, International Statistical Classification of Diseases and Related Health Problems 10th Revision; NA, not applicable.

**Table S11.** Hazard ratios for fatal cardiovascular disease according to BMI-based and clinically defined obesity in status trajectory

| **Organ dysfunction trajectory^a^** | **Event/Total** | **Model^d^ (95% CI)** | ***P* for trend** |
| --- | --- | --- | --- |
| **BMI-based obesity^b^** |  |  |  |
| **No organ dysfunction** |  |  | 0.64 |
| Underweight | 1/2727 | 0.85 (0.12-6.20) |  |
| Normal | 48/140494 | 1.00 (Ref) |  |
| Overweight | 52/170501 | 0.74 (0.50-1.10) |  |
| Obesity | 34/86652 | 0.93 (0.59-1.45) |  |
| **Organ dysfunction only in period 1** |  |  | 0.17 |
| Underweight | 3/170 | NA |  |
| Normal | 3/5890 | 1.00 (Ref) |  |
| Overweight | 13/12139 | 2.15 (0.61-7.62) |  |
| Obesity | 5/10440 | 1.37 (0.32-5.79) |  |
| **Organ dysfunction only in period 2** |  |  | 0.80 |
| Underweight | 0/146 | NA |  |
| Normal | 4/5060 | 1.00 (Ref) |  |
| Overweight | 6/10501 | 6.12 (0.17-2.19) |  |
| Obesity | 6/9278 | 7.44 (0.20-2.71) |  |
| **Persistent organ dysfunction** |  |  | 0.46 |
| Underweight | 0/23 | NA |  |
| Normal | 0/514 | 1.00 (Ref) |  |
| Overweight | 1/1345 | NA |  |
| Obesity | 1/1795 | NA |  |
| **Clinically defined obesity^c^** |  |  |  |
| **No organ dysfunction** |  |  | 0.15 |
| No obesity | 32/136498 | 1.00 (Ref) |  |
| Preclinical obesity | 77/227042 | 1.10 (0.73-1.68) |  |
| Clinical obesity | 26/36834 | 1.53 (0.90-2.61) |  |
| **Organ dysfunction only in period 1** |  |  | 0.54 |
| No obesity | 7/5273 | 1.00 (Ref) |  |
| Preclinical obesity | 12/17825 | 0.88 (0.80-0.97)^e^ |  |
| Clinical obesity | 5/5541 | 1.45 (1.30-1.62)^g^ |  |
| **Organ dysfunction only in period 2** |  |  | 0.50 |
| No obesity | 3/4466 | 1.00 (Ref) |  |
| Preclinical obesity | 10/15457 | 0.73 (0.20-2.70) |  |
| Clinical obesity | 3/5062 | 0.56 (0.11-2.91) |  |
| **Persistent organ dysfunction** |  |  | 0.67 |
| No obesity | 0/428 | 1.00 (Ref) |  |
| Preclinical obesity | 2/2098 | NA |  |
| Clinical obesity | 0/1151 | NA |  |

^a^Organ dysfunction trajectories were constructed from ICD-10 codes across 12 categories defined by the *Lancet Diabetes & Endocrinology* Commission and assessed over two consecutive 2-year intervals. Period 1 began 2 years after the baseline assessment at recruitment (2006–2010), and period 2 for the next consecutive 2 years. Status trajectories were defined by organ dysfunction status across the two periods: participants with no organ dysfunction at both periods were classified as no organ dysfunction; participants with organ dysfunction only in period 1; participants with organ dysfunction only in period 2; participants with organ dysfunction at both periods were classified as persistent organ dysfunction.

^b^BMI-based obesity was defined by BMI. Among non-Asian participants, BMI was classified as underweight (<18.5 kg/m²), normal weight (18.5–24.9 kg/m²), overweight (25.0–29.9 kg/m²), and obese (≥30.0 kg/m²). Among Asian participants, BMI was classified as underweight (<18.5 kg/m²), normal weight (18.5–22.9 kg/m²), overweight (23.0–24.9 kg/m²), and obese (≥25.0 kg/m²).

^c^Clinical obesity was defined as confirmed excess adiposity accompanied by objective evidence of obesity-related organ dysfunctions. For excess obesity, it was defined using a multi-measure anthropometric definition by BMI, WC, WHR, WHtR, and BF. Central adiposity were defined as WC ≥102 cm (men) or ≥88 cm (women) for non-Asian participants and ≥90 cm (men) or ≥80 cm (women) for Asian participants; WHR ≥0.90 (men) or ≥0.85 (women); and WHtR ≥0.50. Elevated BMI was defined as BMI ≥30 kg/m² for non-Asian participants and ≥25 kg/m² for Asian participants. Elevated BF was defined as ≥25% for men and ≥35% for women. Excess adiposity was confirmed if any of the following criteria were met: (1) elevated BF; (2) elevated BMI plus ≥1 elevated central adiposity (WC, WHR, or WHtR); or (3) ≥2 elevated central adiposity. Obesity-related organ dysfunction was defined using ICD-10 diagnosis codes.

^d^Model adjusted hazard ratios by age, sex, deprivation index, smoking status, alcohol consumption, MVPA using Cox regression.

^e^*p*<0.05

^f^*p*<0.01

^g^*p*<.001

Acronyms: WC, waist circumference; WHR,waist-to-hip ratio; WHtR, waist-to-height ratio; BF, body fat percentage; MVPA, moderate-to-vigorous physical activity; BMI, body mass index; ICD-10, International Statistical Classification of Diseases and Related Health Problems 10th Revision; NA, not applicable.

**Table S12.** Hazard ratios for ischemic heart disease according to BMI-based and clinically defined obesity in change trajectory

| **Organ dysfunction trajectory^a^** | **Event/Total** | **Model^d^ (95% CI)** | ***P* for trend** |
| --- | --- | --- | --- |
| **BMI-based obesity^b^** |  |  |  |
| **No organ dysfunction** |  |  | <.001 |
| Underweight | 41/2727 | 1.41 (1.10-1.81)^f^ |  |
| Normal | 1720/140494 | 1.00 (Ref) |  |
| Overweight | 3398/170501 | 1.27 (1.21-1.34)^g^ |  |
| Obesity | 2078/86652 | 1.74 (1.64-1.84)^g^ |  |
| **Increase in organ dysfunction** |  |  | 0.48 |
| Underweight | 7/165 | 1.69 (0.79-3.62) |  |
| Normal | 141/5579 | 1.00 (Ref) |  |
| Overweight | 411/11899 | 1.20 (0.99-1.45) |  |
| Obesity | 333/10458 | 1.14 (0.94-1.40) |  |
| **Decrease in organ dysfunction** |  |  | <.001 |
| Underweight | 5/139 | 1.39 (0.57-3.41) |  |
| Normal | 120/4831 | 1.00 (Ref) |  |
| Overweight | 320/10359 | 1.09 (0.88-1.35) |  |
| Obesity | 363/9502 | 1.44 (1.17-1.78)^g^ |  |
| **Persistent organ dysfunction** |  |  | 0.62 |
| Underweight | 0/11 | NA |  |
| Normal | 11/330 | 1.00 (Ref) |  |
| Overweight | 48/875 | 1.52 (0.79-2.93) |  |
| Obesity | 49/1049 | 1.32 (0.68-2.55) |  |
| **Clinically defined obesity^c^** |  |  |  |
| **No organ dysfunction** |  |  | <.001 |
| No obesity | 1514/136498 | 1.00 (Ref) |  |
| Preclinical obesity | 4388/227042 | 1.41 (1.33-1.49)^g^ |  |
| Clinical obesity | 1335/36834 | 2.39 (2.24-2.55)^g^ |  |
| **Increase in organ dysfunction** |  |  | <.001 |
| No obesity | 110/4960 | 1.00 (Ref) |  |
| Preclinical obesity | 485/17582 | 1.07 (0.87-1.32) |  |
| Clinical obesity | 297/5559 | 1.99 (1.59-2.48) ^g^ |  |
| **Decrease in organ dysfunction** |  |  | <.001 |
| No obesity | 73/4210 | 1.00 (Ref) |  |
| Preclinical obesity | 483/15501 | 1.59 (1.24-2.03)^g^ |  |
| Clinical obesity | 252/5120 | 2.41 (1.85-3.14)^g^ |  |
| **Persistent organ dysfunction** |  |  | 0.05 |
| No obesity | 8/299 | 1.00 (Ref) |  |
| Preclinical obesity | 59/1237 | 1.67 (0.80-3.52) |  |
| Clinical obesity | 41/729 | 2.14 (0.99-4.63) |  |

^a^Organ dysfunction trajectories were constructed from ICD-10 codes across 12 categories defined by the *Lancet Diabetes & Endocrinology* Commission and assessed over two consecutive 2-year intervals. Period 1 began 2 years after the baseline assessment at recruitment (2006–2010), and period 2 for the next consecutive 2 years. Change trajectories were defined by the number of organ dysfunction categories across the two periods: participants with no organ dysfunction at both periods were classified as no organ dysfunction; participants with increased category numbers of organ dysfunction between period 1 and 2 as increase in organ dysfunction; participants with decreased category numbers of organ dysfunction between period 1 and 2 as decrease in organ dysfunction; participants with same number of organ dysfunction categories at both periods were classified as persistent organ dysfunction.

^b^BMI-based obesity was defined by BMI. Among non-Asian participants, BMI was classified as underweight (<18.5 kg/m²), normal weight (18.5–24.9 kg/m²), overweight (25.0–29.9 kg/m²), and obese (≥30.0 kg/m²). Among Asian participants, BMI was classified as underweight (<18.5 kg/m²), normal weight (18.5–22.9 kg/m²), overweight (23.0–24.9 kg/m²), and obese (≥25.0 kg/m²).

^c^Clinical obesity was defined as confirmed excess adiposity accompanied by objective evidence of obesity-related organ dysfunctions. For excess obesity, it was defined using a multi-measure anthropometric definition by BMI, WC, WHR, WHtR, and BF. Central adiposity were defined as WC ≥102 cm (men) or ≥88 cm (women) for non-Asian participants and ≥90 cm (men) or ≥80 cm (women) for Asian participants; WHR ≥0.90 (men) or ≥0.85 (women); and WHtR ≥0.50. Elevated BMI was defined as BMI ≥30 kg/m² for non-Asian participants and ≥25 kg/m² for Asian participants. Elevated BF was defined as ≥25% for men and ≥35% for women. Excess adiposity was confirmed if any of the following criteria were met: (1) elevated BF; (2) elevated BMI plus ≥1 elevated central adiposity (WC, WHR, or WHtR); or (3) ≥2 elevated central adiposity. Obesity-related organ dysfunction was defined using ICD-10 diagnosis codes.

^d^Model adjusted hazard ratios by age, sex, deprivation index, smoking status, alcohol consumption, MVPA using Cox regression.

^e^*p*<0.05

^f^*p*<0.01

^g^*p*<.001

Acronyms: WC, waist circumference; WHR,waist-to-hip ratio; WHtR, waist-to-height ratio; BF, body fat percentage; MVPA, moderate-to-vigorous physical activity; BMI, body mass index; ICD-10, International Statistical Classification of Diseases and Related Health Problems 10th Revision; NA, not applicable.

**Table S13.** Hazard ratios for stroke according to BMI-based and clinically defined obesity in change trajectory

| **Organ dysfunction trajectory^a^** | **Event/Total** | **Model^d^ (95% CI)** | ***P* for trend** |
| --- | --- | --- | --- |
| **BMI-based obesity^b^** |  |  |  |
| **No organ dysfunction** |  |  | <.001 |
| Underweight | 118/2727 | 2.05 (1.79-2.35)^g^ |  |
| Normal | 3608/140494 | 1.00 (Ref) |  |
| Overweight | 5418/170501 | 1.04 (1.00-1.08)^e^ |  |
| Obesity | 3117/86652 | 1.28 (1.23-1.33)^g^ |  |
| **Increase in organ dysfunction** |  |  | 0.06 |
| Underweight | 15/165 | 1.46 (0.87-2.45) |  |
| Normal | 365/5579 | 1.00 (Ref) |  |
| Overweight | 798/11899 | 0.95 (0.84-1.07) |  |
| Obesity | 769/10458 | 1.11 (0.98-1.27) |  |
| **Decrease in organ dysfunction** |  |  | 0.65 |
| Underweight | 15/139 | 1.62 (0.96-2.72) |  |
| Normal | 318/4831 | 1.00 (Ref) |  |
| Overweight | 689/10359 | 0.93 (0.81-1.06) |  |
| Obesity | 645/9502 | 1.04 (0.90-1.19) |  |
| **Persistent organ dysfunction** |  |  | 0.61 |
| Underweight | 2/11 | 2.01 (0.47-8.67) |  |
| Normal | 34/330 | 1.00 (Ref) |  |
| Overweight | 99/875 | 1.00 (0.68-1.49) |  |
| Obesity | 106/1049 | 0.96 (0.65-1.42) |  |
| **Clinically defined obesity^c^** |  |  |  |
| **No organ dysfunction** |  |  | <.001 |
| No obesity | 3066/136498 | 1.00 (Ref) |  |
| Preclinical obesity | 6719/227042 | 1.04 (1.00-1.08)^e^ |  |
| Clinical obesity | 2476/36834 | 1.87 (1.78-1.95)^g^ |  |
| **Increase in organ dysfunction** |  |  | <.001 |
| No obesity | 286/4960 | 1.00 (Ref) |  |
| Preclinical obesity | 1094/17582 | 0.96 (0.85-1.10) |  |
| Clinical obesity | 567/5559 | 1.45 (1.25-1.68)^g^ |  |
| **Decrease in organ dysfunction** |  |  | <.001 |
| No obesity | 223/4210 | 1.00 (Ref) |  |
| Preclinical obesity | 967/15501 | 1.05 (0.91-1.22) |  |
| Clinical obesity | 477/5120 | 1.47 (1.25-1.73)^g^ |  |
| **Persistent organ dysfunction** |  |  | 0.006 |
| No obesity | 34/299 | 1.00 (Ref) |  |
| Preclinical obesity | 94/1237 | 0.57 (0.38-0.84)^f^ |  |
| Clinical obesity | 113/729 | 1.16 (0.78-1.73) |  |

^a^Organ dysfunction trajectories were constructed from ICD-10 codes across 12 categories defined by the *Lancet Diabetes & Endocrinology* Commission and assessed over two consecutive 2-year intervals. Period 1 began 2 years after the baseline assessment at recruitment (2006–2010), and period 2 for the next consecutive 2 years. Change trajectories were defined by the number of organ dysfunction categories across the two periods: participants with no organ dysfunction at both periods were classified as no organ dysfunction; participants with increased category numbers of organ dysfunction between period 1 and 2 as increase in organ dysfunction; participants with decreased category numbers of organ dysfunction between period 1 and 2 as decrease in organ dysfunction; participants with same number of organ dysfunction categories at both periods were classified as persistent organ dysfunction.

^b^BMI-based obesity was defined by BMI. Among non-Asian participants, BMI was classified as underweight (<18.5 kg/m²), normal weight (18.5–24.9 kg/m²), overweight (25.0–29.9 kg/m²), and obese (≥30.0 kg/m²). Among Asian participants, BMI was classified as underweight (<18.5 kg/m²), normal weight (18.5–22.9 kg/m²), overweight (23.0–24.9 kg/m²), and obese (≥25.0 kg/m²).

^c^Clinical obesity was defined as confirmed excess adiposity accompanied by objective evidence of obesity-related organ dysfunctions. For excess obesity, it was defined using a multi-measure anthropometric definition by BMI, WC, WHR, WHtR, and BF. Central adiposity were defined as WC ≥102 cm (men) or ≥88 cm (women) for non-Asian participants and ≥90 cm (men) or ≥80 cm (women) for Asian participants; WHR ≥0.90 (men) or ≥0.85 (women); and WHtR ≥0.50. Elevated BMI was defined as BMI ≥30 kg/m² for non-Asian participants and ≥25 kg/m² for Asian participants. Elevated BF was defined as ≥25% for men and ≥35% for women. Excess adiposity was confirmed if any of the following criteria were met: (1) elevated BF; (2) elevated BMI plus ≥1 elevated central adiposity (WC, WHR, or WHtR); or (3) ≥2 elevated central adiposity. Obesity-related organ dysfunction was defined using ICD-10 diagnosis codes.

^d^Model adjusted hazard ratios by age, sex, deprivation index, smoking status, alcohol consumption, MVPA using Cox regression.

^e^*p*<0.05

^f^*p*<0.01

^g^*p*<.001

Acronyms: WC, waist circumference; WHR,waist-to-hip ratio; WHtR, waist-to-height ratio; BF, body fat percentage; MVPA, moderate-to-vigorous physical activity; BMI, body mass index; ICD-10, International Statistical Classification of Diseases and Related Health Problems 10th Revision; NA, not applicable.

**Table S14.** Hazard ratios for fatal cardiovascular disease according to BMI-based and clinically defined obesity in change trajectory

| **Organ dysfunction trajectory^a^** | **Event/Total** | **Model^d^ (95% CI)** | ***P* for trend** |
| --- | --- | --- | --- |
| **BMI-based obesity^b^** |  |  |  |
| **No organ dysfunction** |  |  | 0.64 |
| Underweight | 1/2727 | 0.85 (0.12-6.20) |  |
| Normal | 48/140494 | 1.00 (Ref) |  |
| Overweight | 52/170501 | 0.74 (0.50-1.10) |  |
| Obesity | 34/86652 | 0.93 (0.59-1.45) |  |
| **Increase in organ dysfunction** |  |  | 0.15 |
| Underweight | 3/165 | NA |  |
| Normal | 3/5579 | 1.00 (Ref) |  |
| Overweight | 13/11899 | 2.10 (0.59-7.44) |  |
| Obesity | 5/10458 | 1.31 (0.31-5.55) |  |
| **Decrease in organ dysfunction** |  |  | 0.75 |
| Underweight | 0/139 | NA |  |
| Normal | 4/4831 | 1.00 (Ref) |  |
| Overweight | 6/10359 | 0.60 (0.17-2.16) |  |
| Obesity | 6/9502 | 0.71 (0.19-2.58) |  |
| **Persistent organ dysfunction** |  |  | 0.18 |
| Underweight | 0/11 | NA |  |
| Normal | 0/330 | 1.00 (Ref) |  |
| Overweight | 1/875 | NA |  |
| Obesity | 1/1049 | NA |  |
| **Clinically defined obesity^c^** |  |  |  |
| **No organ dysfunction** |  |  | 0.15 |
| No obesity | 32/136498 | 1.00 (Ref) |  |
| Preclinical obesity | 77/227042 | 1.10 (0.73-1.68) |  |
| Clinical obesity | 26/36834 | 1.53 (0.90-2.61) |  |
| **Increase in organ dysfunction** |  |  | 0.51 |
| No obesity | 7/4960 | 1.00 (Ref) |  |
| Preclinical obesity | 12/17582 | 0.52 (0.20-1.33) |  |
| Clinical obesity | 5/5559 | 0.71 (0.22-2.29) |  |
| **Decrease in organ dysfunction** |  |  | 0.48 |
| No obesity | 3/4210 | 1.00 (Ref) |  |
| Preclinical obesity | 10/15501 | 0.70 (0.19-2.59) |  |
| Clinical obesity | 3/5120 | 0.55 (0.11-2.81) |  |
| **Persistent organ dysfunction** |  |  | 0.58 |
| No obesity | 0/299 | 1.00 (Ref) |  |
| Preclinical obesity | 2/1237 | NA |  |
| Clinical obesity | 0/729 | NA |  |

^a^Organ dysfunction trajectories were constructed from ICD-10 codes across 12 categories defined by the *Lancet Diabetes & Endocrinology* Commission and assessed over two consecutive 2-year intervals. Period 1 began 2 years after the baseline assessment at recruitment (2006–2010), and period 2 for the next consecutive 2 years. Change trajectories were defined by the number of organ dysfunction categories across the two periods: participants with no organ dysfunction at both periods were classified as no organ dysfunction; participants with increased category numbers of organ dysfunction between period 1 and 2 as increase in organ dysfunction; participants with decreased category numbers of organ dysfunction between period 1 and 2 as decrease in organ dysfunction; participants with same number of organ dysfunction categories at both periods were classified as persistent organ dysfunction.

^b^BMI-based obesity was defined by BMI. Among non-Asian participants, BMI was classified as underweight (<18.5 kg/m²), normal weight (18.5–24.9 kg/m²), overweight (25.0–29.9 kg/m²), and obese (≥30.0 kg/m²). Among Asian participants, BMI was classified as underweight (<18.5 kg/m²), normal weight (18.5–22.9 kg/m²), overweight (23.0–24.9 kg/m²), and obese (≥25.0 kg/m²).

^c^Clinical obesity was defined as confirmed excess adiposity accompanied by objective evidence of obesity-related organ dysfunctions. For excess obesity, it was defined using a multi-measure anthropometric definition by BMI, WC, WHR, WHtR, and BF. Central adiposity were defined as WC ≥102 cm (men) or ≥88 cm (women) for non-Asian participants and ≥90 cm (men) or ≥80 cm (women) for Asian participants; WHR ≥0.90 (men) or ≥0.85 (women); and WHtR ≥0.50. Elevated BMI was defined as BMI ≥30 kg/m² for non-Asian participants and ≥25 kg/m² for Asian participants. Elevated BF was defined as ≥25% for men and ≥35% for women. Excess adiposity was confirmed if any of the following criteria were met: (1) elevated BF; (2) elevated BMI plus ≥1 elevated central adiposity (WC, WHR, or WHtR); or (3) ≥2 elevated central adiposity. Obesity-related organ dysfunction was defined using ICD-10 diagnosis codes.

^d^Model adjusted hazard ratios by age, sex, deprivation index, smoking status, alcohol consumption, MVPA using Cox regression.

^e^*p*<0.05

^f^*p*<0.01

^g^*p*<.001

Acronyms: WC, waist circumference; WHR,waist-to-hip ratio; WHtR, waist-to-height ratio; BF, body fat percentage; MVPA, moderate-to-vigorous physical activity; BMI, body mass index; ICD-10, International Statistical Classification of Diseases and Related Health Problems 10th Revision; NA, not applicable.

**Figure S1.** Comparison adjusted hazard ratios for BMI-based and clinically defined obesity in relation to incident MACE


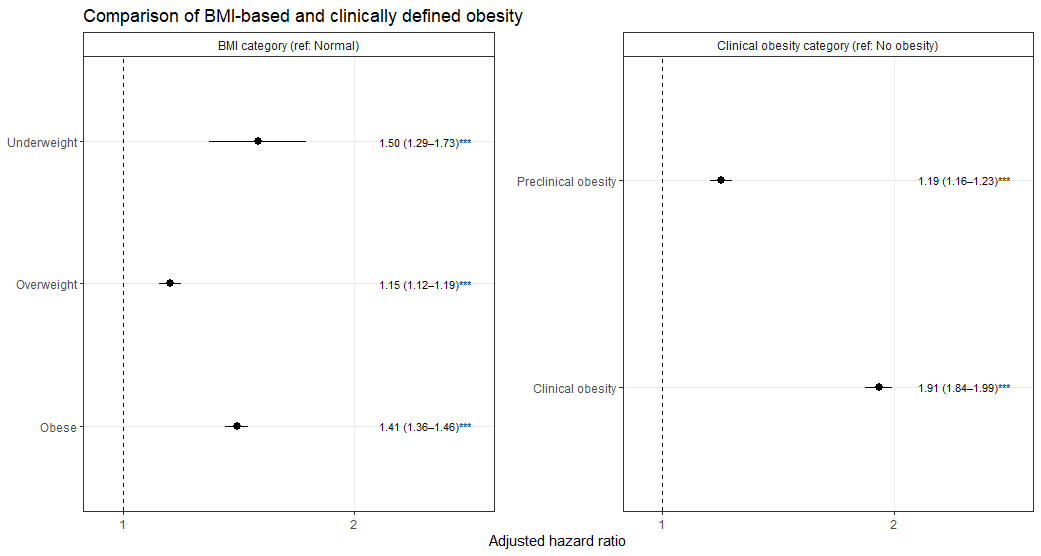


Hazard ratios and 95% confidence intervals were estimated using Cox proportional hazards models fitted in the same analytic sample. The BMI-based obesity model was adjusted for age at interview start, sex, moderate-to-vigorous physical activity, alcohol consumption, Townsend deprivation index, and smoking status, with normal weight as the reference category. The clinically defined obesity model was adjusted for the same covariates, with no obesity as the reference category. Values shown to the right of each point represent the adjusted hazard ratio and 95% confidence interval. Asterisks indicate statistical significance.

^*^*p*<0.05

^**^*p*<0.01

^***^*p*<.001

**Acronyms:** BMI, body mass index; CI, confidence interval; HR, hazard ratio; MACE, major adverse cardiovascular events.

**Figure S2.** Calibration plot for the BMI category model at 10 years.


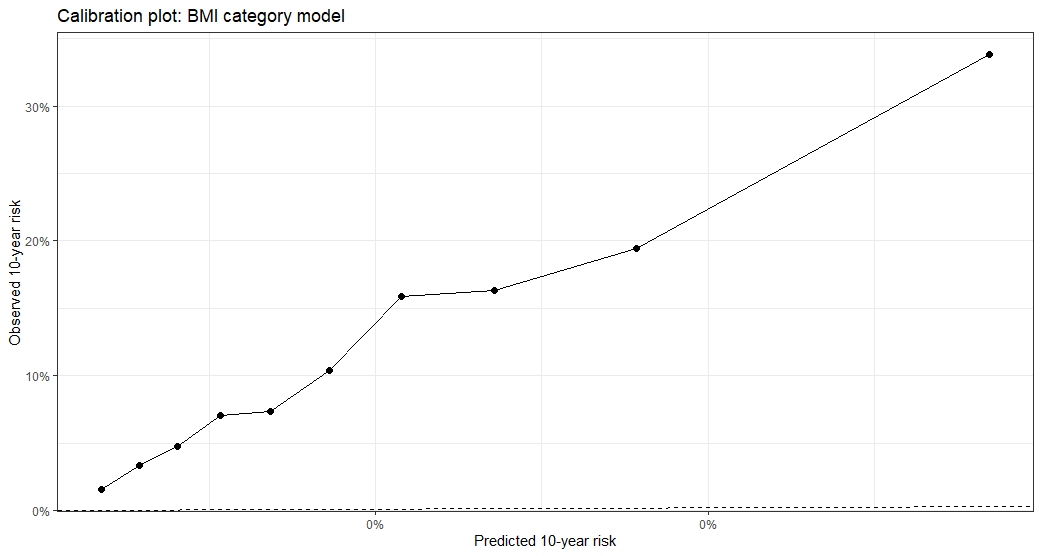


The plot compares predicted 10-year risk and observed 10-year risk of major adverse cardiovascular events across deciles of predicted risk for the Cox model including BMI category. The model was adjusted for age at interview start, sex, moderate-to-vigorous physical activity, alcohol consumption, Townsend deprivation index, and smoking status. The diagonal line indicates perfect agreement between predicted and observed risk.

**Acronyms:** BMI, body mass index; MACE, major adverse cardiovascular events.

**Figure S3.** Calibration plot for the clinical obesity model at 10 years.


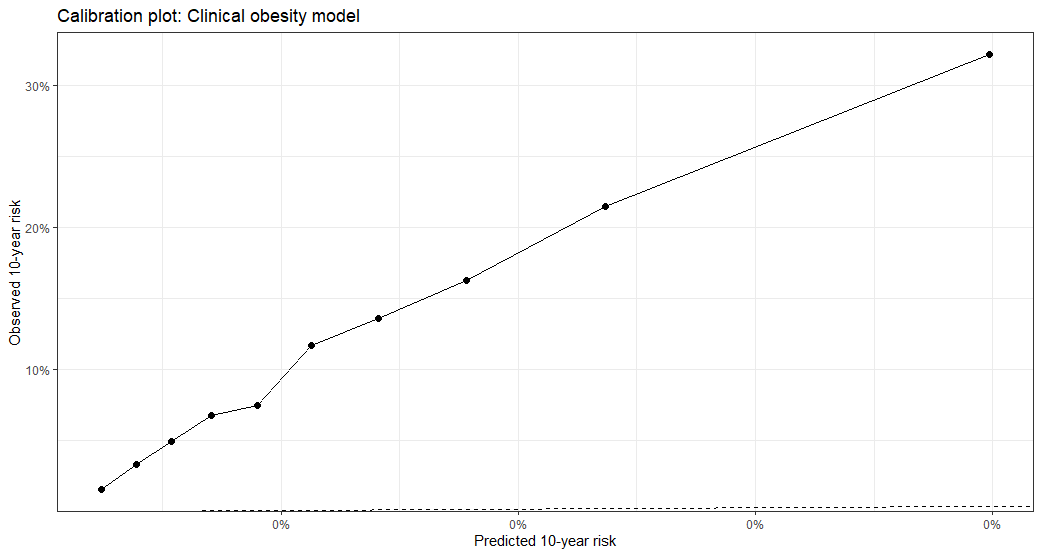


The plot compares predicted 10-year risk and observed 10-year risk of major adverse cardiovascular events across deciles of predicted risk for the Cox model including clinically defined obesity category. The model was adjusted for age at interview start, sex, moderate-to-vigorous physical activity, alcohol consumption, Townsend deprivation index, and smoking status. The diagonal line indicates perfect agreement between predicted and observed risk.

**Acronyms:** MACE, major adverse cardiovascular events.
