# Supplementary material for: Association of organ dysfunction trajectories and major adverse cardiovascular events using clinical obesity in UK Biobank
Source: Front Endocrinol (Lausanne). 2026 May 14;17:1844870. doi: 10.3389/fendo.2026.1844870 (PMC13215929; doi:10.3389/fendo.2026.1844870)
Supplement: Supplementary file 2 [file Presentation1.pptx]

## Slide 1
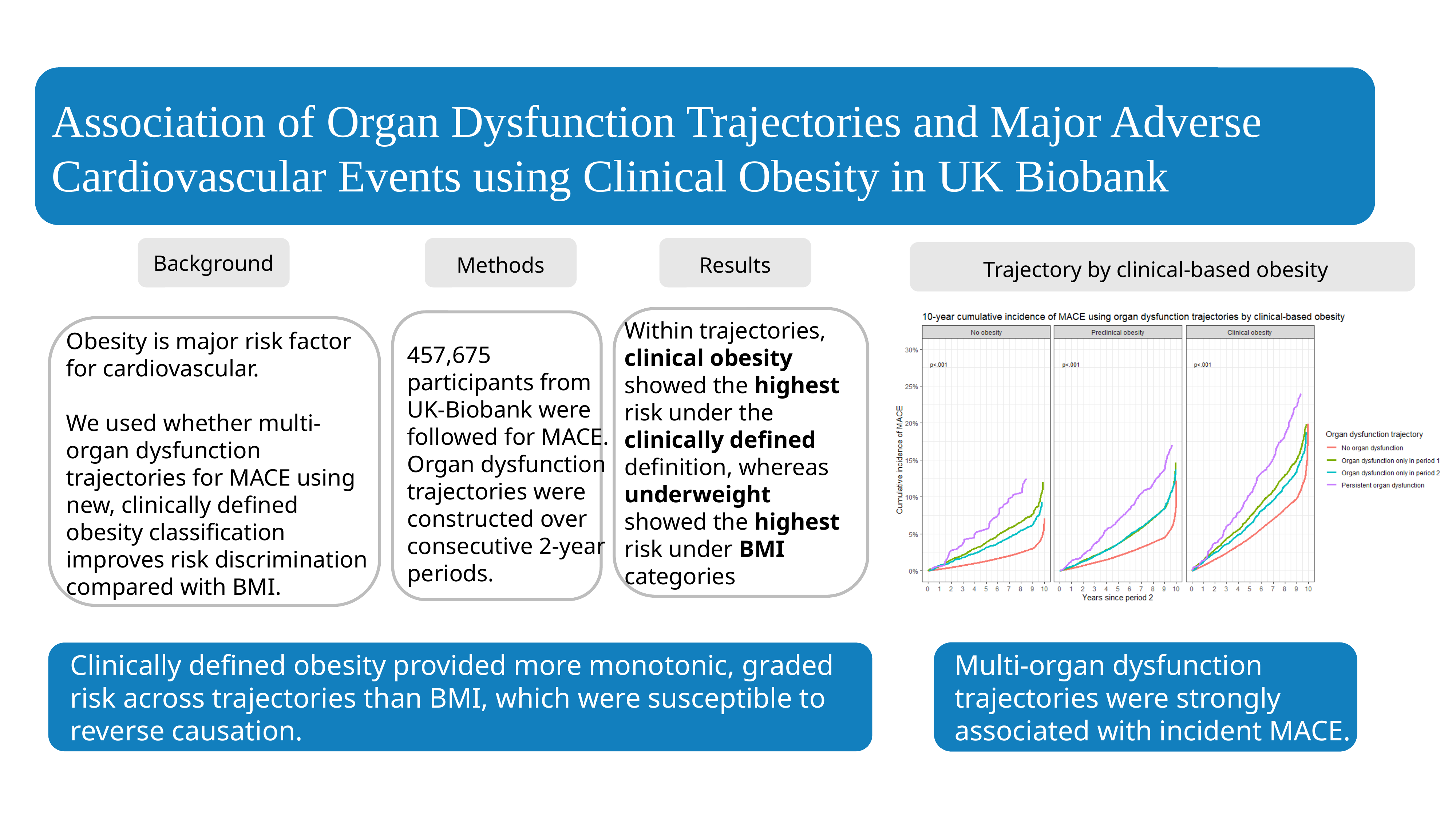

Association of Organ Dysfunction Trajectories and Major Adverse Cardiovascular Events using Clinical Obesity in UK Biobank
Background
Methods
Results
Trajectory by clinical-based obesity
Obesity is major risk factor for cardiovascular.
We used whether multi-organ dysfunction trajectories for MACE using new, clinically defined obesity classification improves risk discrimination compared with BMI.
457,675 participants from UK-Biobank were followed for MACE.
Organ dysfunction trajectories were constructed over consecutive 2-year periods.
Within trajectories, clinical obesity showed the highest risk under the clinically defined definition, whereas underweight showed the highest risk under BMI categories
Multi-organ dysfunction trajectories were strongly associated with incident MACE.
Clinically defined obesity provided more monotonic, graded risk across trajectories than BMI, which were susceptible to reverse causation.
